# Importance of the RNA secondary structure for the relative accumulation of clustered viral microRNAs

**DOI:** 10.1093/nar/gku424

**Published:** 2014-05-15

**Authors:** Maud Contrant, Aurélie Fender, Béatrice Chane-Woon-Ming, Ramy Randrianjafy, Valérie Vivet-Boudou, Delphine Richer, Sébastien Pfeffer

**Affiliations:** Architecture et Réactivité de l’ARN – UPR 9002, Institut de Biologie Moléculaire et Cellulaire du CNRS, Université de Strasbourg, 15 rue René Descartes, F-67084 Strasbourg Cedex, France

## Abstract

Micro (mi)RNAs are small non-coding RNAs with key regulatory functions. Recent advances in the field allowed researchers to identify their targets. However, much less is known regarding the regulation of miRNAs themselves. The accumulation of these tiny regulators can be modulated at various levels during their biogenesis from the transcription of the primary transcript (pri-miRNA) to the stability of the mature miRNA. Here, we studied the importance of the pri-miRNA secondary structure for the regulation of mature miRNA accumulation. To this end, we used the Kaposi's sarcoma herpesvirus, which encodes a cluster of 12 pre-miRNAs. Using small RNA profiling and quantitative northern blot analysis, we measured the absolute amount of each mature miRNAs in different cellular context. We found that the difference in expression between the least and most expressed viral miRNAs could be as high as 60-fold. Using high-throughput selective 2′-hydroxyl acylation analyzed by primer extension, we then determined the secondary structure of the long primary transcript. We found that highly expressed miRNAs derived from optimally structured regions within the pri-miRNA. Finally, we confirmed the importance of the local structure by swapping stem-loops or by targeted mutagenesis of selected miRNAs, which resulted in a perturbed accumulation of the mature miRNA.

## INTRODUCTION

Kaposi's sarcoma associated herpesvirus (KSHV) is a human gammaherpesvirus associated to oncogenic disorders, such as Kaposi's sarcoma, B-lymphomas or Castleman disease (1). Similarly to other herpesviruses, KSHV was shown to encode several microRNAs (miRNAs) deriving from 12 precursors. Interestingly, all KSHV pre-miRNAs are expressed as a polycistronic transcript from the same genomic region, which is associated to latency (2–4). Ten of them are in an intron (pre-miR-K1 to -K9 and pre-miR-K11) between K12 and v-FLIP open reading frames (ORFs), whereas pre-miR-K10 and -K12 are in the coding sequence and the 3′ UTR of K12, respectively. All KSHV miRNAs are expressed during latency from the latent promoter located upstream of ORF73 (5). In addition, miR-K10 and miR-K12 are also induced during the lytic phase (2), and a recent study showed that they are all present at significant levels upon viral reactivation (6). Furthermore, they were detected in virions (7), indicating that they might play a role in the very early stages of infection.

Numerous functions, such as regulation of the immune response, cell cycle or apoptosis, have been attributed to KSHV miRNAs (see Ref. (8) for a recent review). However, much less is known regarding the regulation of expression of these viral products. In animals, miRNAs derive from a large primary transcript (pri-miRNA) that contains a unique or several miRNA precursors (pre-miRNA), structured in a stem-loop. The pri-miRNA is first processed in the nucleus by the RNase III enzyme Drosha, associated to its cofactor DGCR8, to release the pre-miRNA. After export into the cytoplasm, it is further processed by another RNase III enzyme, Dicer, associated to TAR recognition binding protein (TRBP), to generate the miRNA/miRNA* duplex. Upon unwinding, one of the two strands of the duplex is assembled into the RNA-induced silencing complex to modulate the expression of target mRNAs (reviewed in (9)). KSHV miRNAs, like the vast majority of miRNAs of viral origin, are expressed following this canonical pathway. A large proportion (up to 40%) of animal miRNAs are expressed in clusters (10), and interestingly this is also the case for gammaherpesviruses’ miRNAs. Even though clustered miRNAs all derive from the same primary transcript, there is increasing evidence that the end products, i.e. miRNA mature sequences, do not accumulate to similar levels. This could be due to the regulation of a number of steps during the processing of the pri-miRNA into mature miRNA. Thus, some have reported that Drosha and DGCR8 activity could be modulated by specific cofactors, such as, for example, hnRNPA1. Indeed, this protein was shown to bind specifically to pre-miR-18a within the pri-mir-17∼92 cluster and to facilitate its processing, therefore, resulting in an increased expression of miR-18a (11). Alternatively, the processing of the pri-miRNA can also be modulated by structural determinants. In particular, researchers showed that the apical loop and the junction between the basal stem and the flanking segments of the stem-loop structure are crucial determinants for Drosha/DGCR8 recognition and cleavage (12–14). Moreover, the tertiary structure of long pri-miRNAs may affect the accessibility of stem-loops for Drosha/DGCR8 recognition (15,16). Finally, the processing of the pre-miRNA by Dicer is also affected by the RNA structure (see Ref. (17) for a review).

Here, we investigated the importance of RNA secondary structure of the long primary transcript containing the 10 intronic miRNAs from KSHV (pri-miR-K10/12) for the accumulation of mature miRNAs. Using deep sequencing and quantitative northern blot analysis, we first confirmed that the viral miRNAs accumulate to dramatically different levels; this phenomenon was dependent on the cell line tested. Using plasmids expressing either the complete primary transcript or only each individual miRNA separately, we also observed that the level of miRNA expression was dramatically influenced by the genomic context. Then, the secondary structure of pri-miR-K10/12 was determined in solution by taking advantage of high-throughput selective 2′-hydroxyl acylation analyzed by primer extension (hSHAPE) (18). This method is very powerful to assess the structure of long RNA, at every single nucleotide, as exemplified by the resolution of the 2D model of the entire HIV-1 genomic RNA (19). We could classify stem-loop structures embedding miRNAs into sub- or optimally folded ones for Drosha/DGCR8 recognition and cleavage. This correlated well with the expression level of the corresponding mature miRNAs in cells and was confirmed by a targeted mutational analysis. Altogether, we demonstrate that the RNA structure significantly governs the net accumulation of mature miRNAs in cells.

## MATERIALS AND METHODS

### Cell lines and culture conditions

All B-cell lines were cultured in a humidified 5% CO_2_ atmosphere at 37°C in RPMI (Roswell Park Memorial Institute) 1640 medium containing 10% fetal calf serum. DG-75-EGFP or DG-75-K10/12 were grown in the presence of 7.5 μg/ml blasticidin (for gene insert maintenance). BC-3 growth medium was supplemented with 50 μM mercaptoethanol. DG-75-EGFP and DG-75-K10/12 stably express either EGFP protein or pri-miR-K10/12 comprising the 10 intronic pre-miRNAs (pre-miR-K1 to -K9 and pre-miR-K11, Supplementary Figure S1A) from KSHV and were generated previously (20,21).

### RNA preparation

Total RNA was extracted from cells using Tri-reagent (MRC, Inc).

The pri-miR-K10/12, derived from BCBL-1 cell line, was transcribed from PCR-generated DNA templates carrying a T7 promoter. For primer sequences see Supplementary Table S1. *In vitro* RNA synthesis was done by T7 RNA polymerase (Ambion) and pri-miR-K10/12 transcript (3157 nt) was purified on a 0.8% low-melting agarose gel. The band of interest was melted at 65°C during 10 min, and after 5 min at 42°C, the RNA was eluted using 1 U of agarase 1 (Thermo Scientific) during 40 min at 42°C. After phenol/chloroform/isoamyl alcohol (PCI) extraction and ethanol precipitation, the RNAs were pelleted and recovered in MilliQ water.

### Deep sequencing

#### Small RNA library preparation

Libraries were prepared as previously described with some modifications (22). Briefly, 10 μg of total RNA from BCBL-1, BC-3 and DG-75-K10/12 were size fractionated on a 17.5% urea denaturing polyacrylamide gel. The 19–33 nt band was excised, crushed and solved in 2 volumes of 0.3 M NaCl by overnight agitation at 4°C. Small RNAs were sequentially ligated to 3′ adapter (5′P-NNNNTGGAATTCTCGGGTGCCAAGG-[C7amino]) and then to 5′ adapter (GUUCAGAGUUCUACAGUCCACGAUCNNNN) (with truncated T4 RNA Ligase 2 and T4 RNA Ligase 1, respectively). Four degenerate bases were included at the 3′ end of the 5′ adapter and the 5′ end of the 3′ adapter to minimize ligation bias due to secondary structure formation (23,24). 3′ ligation was done overnight at 16°C, in 12.5% PEG 8000 to optimize ligation. Prior to this, the 3′ adapter was adenylated using the 5′ DNA adenylation kit according to the manufacturer's recommandations (New England Biolab, #E2610S). After each ligation step the product was purified in denaturing polyacrylamide gel. In order to generate cDNA libraries, RNA was reverse transcribed with SuperScript III RT (Invitrogen) and primer GCCTGGCACCCGAGAATTCCA, then amplified by PCR with forward primer AATGATACGGCGACCACCGAGATCTACACGTTCAGAGTTCTACAGTCCGA and different reverse primers for multiplex sequencing (RPI1, RPI4 and RPI5, Illumina PCR primers) during 14 or 17 cycles (for BC-3, or DG-75-K10/12 and BCBL-1 libraries, respectively) (45″ at 94°C, 85″ at 50°C and 60″ at 72°C). PCR products were sent for large-scale sequencing. Small RNA sequencing was performed by the IGBMC Microarray and Sequencing platform, member of the France Genomique program, using an Illumina HiSeq 2000 instrument with a read length of 50 nt.

#### Deep sequencing data analysis

Sequencing reads were preprocessed and annotated using a set of custom Python scripts encompassing different tools. First of all, the Dustmasker program (25) and FASTX-Toolkit (http://hannonlab.cshl.edu/fastx_toolkit) were successively applied to filter out low complexity reads and remove instances of the 3′ adapter. Degenerate bases incorporated during the library preparation protocol were also trimmed at this step. Remaining reads ≥15 nt in length were then mapped simultaneously to the human (assembly version hg19 – UCSC repository) and KSHV (accession number NC_009333.1 – RefSeq database) genomes using Bowtie 0.12.7 (26), permitting up to 2 mismatches in total with no more than 1 mismatch in the first 15 nucleotides of each read. Only alignments from the lowest mismatch stratum were recorded and reads that could map to more than 50 loci were discarded.

To obtain a global overview of each library, several non-coding RNA families were successively annotated using BEDTools 2.16.2 (27) by comparing their genomic coordinates to those of the aligned reads, and by keeping reads with at least 80% of their length inside the genomic feature. Sequences coming from known *Homo sapiens* and KSHV miRNAs (miRBase registry v.19, (28)) were thus identified, followed by those deriving from human rRNAs, tRNAs, snRNAs, snoRNAs and miscRNAs (UCSC (University of California, Santa Cruz) repository or Ensembl Release 68). During the quantification process, multiple mapped reads were weighted by the number of mapping sites in genomic features of interest. It must be noted that prior to this analysis, genomic coordinates of human and KSHV miRNAs were verified by aligning corresponding pre-miRNA and mature miRNA sequences (miRBase v.19) to the genomes used during the mapping step. Furthermore, two groups of hypothetical viral genomic features, antisense miRNAs (AS-miRs) and miRNA offset RNAs (moRs), were created and submitted to the same annotation process. KHSV AS-miRs were defined as opposite sequences to known miRNAs while moR-5p and moR-3p were defined as being the immediate 20 nt long flanking sequences surrounding the mature forms of each KSHV pre-miRNA.

Finally, less stringent mapping parameters were applied for the detailed analysis of KSHV miRNA isoforms. Indeed, reads with up to 9 mismatches in total were allowed in order to detect more miRNA altered forms: full length, 3′-trimmed, 3′-tailed, 5′-trimmed, 5′-tailed and every possible combination (5′-trimmed + 3′-trimmed, 5′-trimmed + 3′-tailed, 5′-tailed + 3′-trimmed and 5′-tailed + 3′-tailed); whether they are identical or different to the template sequence.

The data discussed in this publication have been deposited in NCBI's (National Center for Biotechnology Information) Gene Expression Omnibus (GEO) (29) and will be accessible through GEO Series accession number GSE53296.

### Northern blot analysis

Northern blot analyses were performed as described before (30). Briefly, after extraction using Tri-reagent (MRC, Inc), total RNA was separated on a 15% urea-acrylamide gel. After transfer on a nylon membrane (HybondNX, Amersham Biosciences) in MilliQ water, RNAs were crosslinked to the membrane by chemical treatment at 60°C using 1-ethyl-3-[3-dimethylaminopropyl]carbodiimide hydrochloride (EDC) (Sigma) for 1 h 30 min. RNAs were detected with specific 5′-^32^P labeled oligonucleotides (Supplementary Table S1). The signals were quantified using a Fuji Bioimager FLA5100. For quantification, an RNA oligonucleotide (IDT) identical to the miRNA tested, along with 5 μg of total yeast RNA (Ambion) was loaded at increasing concentration (from 0.1 to 10 fmol). A standard curve was generated by plotting the signal intensity against the amount of RNA oligonucleotides loaded, and was used to calculate the absolute amount of miRNAs in the different cell lines tested. miR-16 was probed as a loading control.

### SHAPE analysis

#### RNA probing

One pmol of purified RNA was denatured 3 min at 95°C, cooled on ice 3 min and folded in Cacodylate buffer (10 mM sodium cacodylate pH 6.8, 10 mM NaCl, 10 mM MgCl_2_, 0.1 mM ethylenediaminetetraacetic acid (EDTA)) for SHAPE experiments (31,32) or folded in 10x structure buffer provided by Ambion for RNase T1 experiments, during 30 min at 37°C. After folding, 1 μg of yeast total RNA (Ambion) was added and the mix was incubated with 100 mM of benzoyl cyanide (BzCN) in a final volume of 10 μl during 1 min for chemical modification, or treated 15 or 25 min at 25°C with 0.05 U of RNase T1. BzCN was inactivated by addition of 90 μl MilliQ water and enzymatic reactions were stopped with 5 μl of 0.1 M EDTA. The modified RNAs were PCI extracted and ethanol precipitated. Control reactions of BzCN modification were treated with anhydrous Dimethyl sulfoxide (DMSO). Pelleted RNAs were resuspended in 7 μl of 1x TE buffer (10 mM Tris-HCl pH 7.5, 1 mM EDTA) before retrotranscription. Experiments were done in triplicate with different preparation of RNA transcripts.

#### Primer extension

Thirteen 21–24 nt long DNA primers (Life Technologies) were used for primer reverse transcription reactions (Supplementary Table S1 and Supplementary Figure S1B). Modified RNAs were denatured for 3 min at 95°C then cooled down on ice for 3 min. Reverse transcription reactions were performed according to Aktar *et al.*, with some modifications (33). Briefly, retrotranscription were done with 0.9 pmol of 5′ fluorophore labeled primer (6-FAM for the treated sample and VIC® for negative control), 484 μM dNTP, 1x RTB (Life Science), 2 U AMV RT enzyme (Life Science), 2 min at 42°C, 30 min at 50°C and 10 min at 60°C. In parallel, two sequencing reactions were done with 1 pmol of non-treated RNAs (per reaction), using ddATP and NED® labeled primer, or ddGTP and positron emission tomography labeled primer. ddNTP were at 5.56 μM and dNTP at 42 μM. All four reverse transcription reactions were pooled, then cDNAs were purified by phenol/chloroform extraction, ethanol precipitated and finally dissolved in 10 μl deionized formamide.

#### SHAPE Data processing

Fluorophore labeled cDNAs were resolved by capillary electrophoresis using ABI 3130xl Genetic Analyzer, capillary size 50 cm. The resulting electropherograms were converted into SHAPE reactivities using SHAPE Finder software and normalized (34). This procedure has been described in detail in Athavale *et al.* (35). For the normalization of SHAPE reactivities, the 2% highest values from the pool were removed. Then the remaining 8% of the highest values were averaged and all reactivities were divided by this average value.

#### RNA secondary structure prediction and modelisation

We folded the entire cluster RNA (3157 nt) using the thermodynamics-based free energy minimization algorithm in the RNAstructure software package, version 5.3 (36). For the minimum free energy structure, we used the pseudo-energy constraints specific for SHAPE data with default values for the SHAPE slope (2.6 kcal/mol) and SHAPE intercept (−0.8 kcal/mol). The maximum pairing distances were set at 200 nucleotides. The final structure of pri-miR-K10/12 was drawn with Assemble 2 program, available at http://bioinformatics.org/assemble (37).

### miRNA expression tests and mutagenesis

Plasmid pcDNA-K10/12, derived from pcDNA5 (Invitrogen) and containing the wild type (wt) pri-miR-K10/12, and plasmids expressing individual miRNAs pcDNA-K1, K2, K3, K4, K6, K7, K8, K9 and K11 have been described before (21).

Plasmid pcDNA-K10/12 was mutated using the Phusion site-directed mutagenesis kit (Thermo Scientific) and transformation of *Escherichia coli* TOP10 strain. Positive clones were identified by sequencing (GATC Biotech, France). Four swapping mutants were designed: mutant mut1 with swapped miR-K9- and miR-K6 stem-loops (minus the single-stranded flanking segments); mutant mut2 with swapped miR-K9- and miR-K11 stem-loops (minus the single-stranded flanking segments); mutant mut3 with swapped pre-miR-K5 and pre-miR-K6; mutant mut4 with swapped pre-miR-K5 and pre-miR-K11. Thirteen mutants termed mutA to mutM with various changes in the pre-miRNA sequence of miR-K2, K5 and K9 were designed. See text and Figure 6 and Supplementary Figure S6 for more details. Expression tests were conducted as follow: 2 μg of plasmids (wt and mutants mut1 to 4) or 0.9 μg of plasmids (wt and mutants mutA to M) were used to transfect HEK293Grip cells per 6 well/plate (mut1 to 4) or 12 well/plate (mutA to M). Total RNA was collected after 48 h and miRNA expression was analyzed by northern blot, using 5 μg of total RNA (mut1 to 4) or 7 μg of total RNA (mutA to M) and standard protocol. miR-16 was probed as a loading control and used for signal normalization.

**Figure 1.**
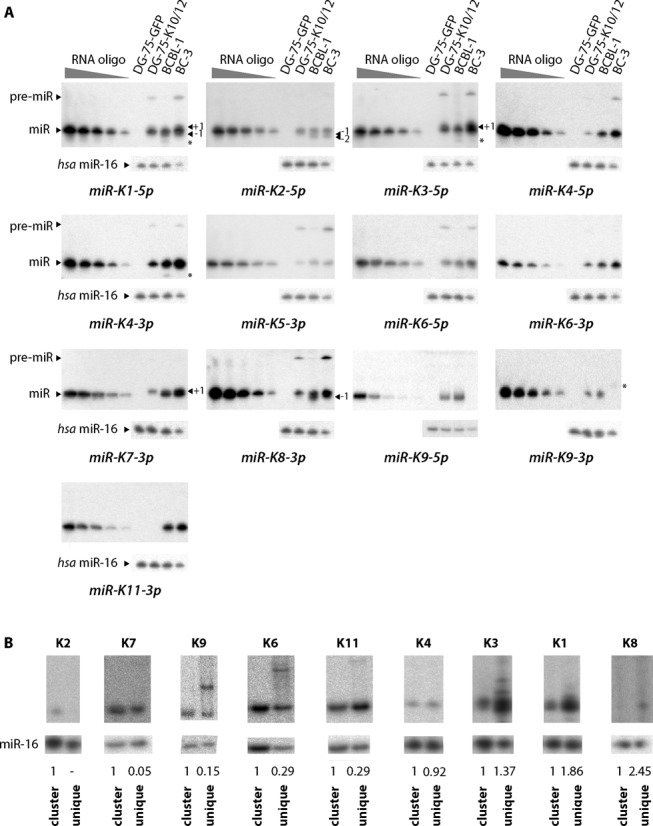
Absolute quantification of mature miRNAs from KSHV in various cell lines. (**A**) To quantify the absolute amount of each miRNA in the different cell types, a standard RNA oligonucleotide, identical to the tested miRNA, was loaded on the same gel at increasing amounts (0.1–10 fmol) along with 5 μg (DG-75-EGFP, DG-75-K10/12 and BCBL-1) or 2.5 μg (BC-3) of total RNA. Representative northern blots for each of the miRNA tested (*n* = 3–4) are shown. As a negative control, total RNA from a non-infected cell-line DG-75-EGFP was used. miR-16 was probed as a loading control. (**B**) pcDNA5 expression plasmids with the entire miRNA cluster (cluster) or only single pre-miRNA (unique) were transfected in HEK293Grip cells and total RNA analyzed by northern blot for the indicated miRNAs. miR-16 was probed as a loading control, and the KSHV miRNA signals quantified and normalized relative to the cluster-transfected cells, which was set to 1.

**Figure 2.**
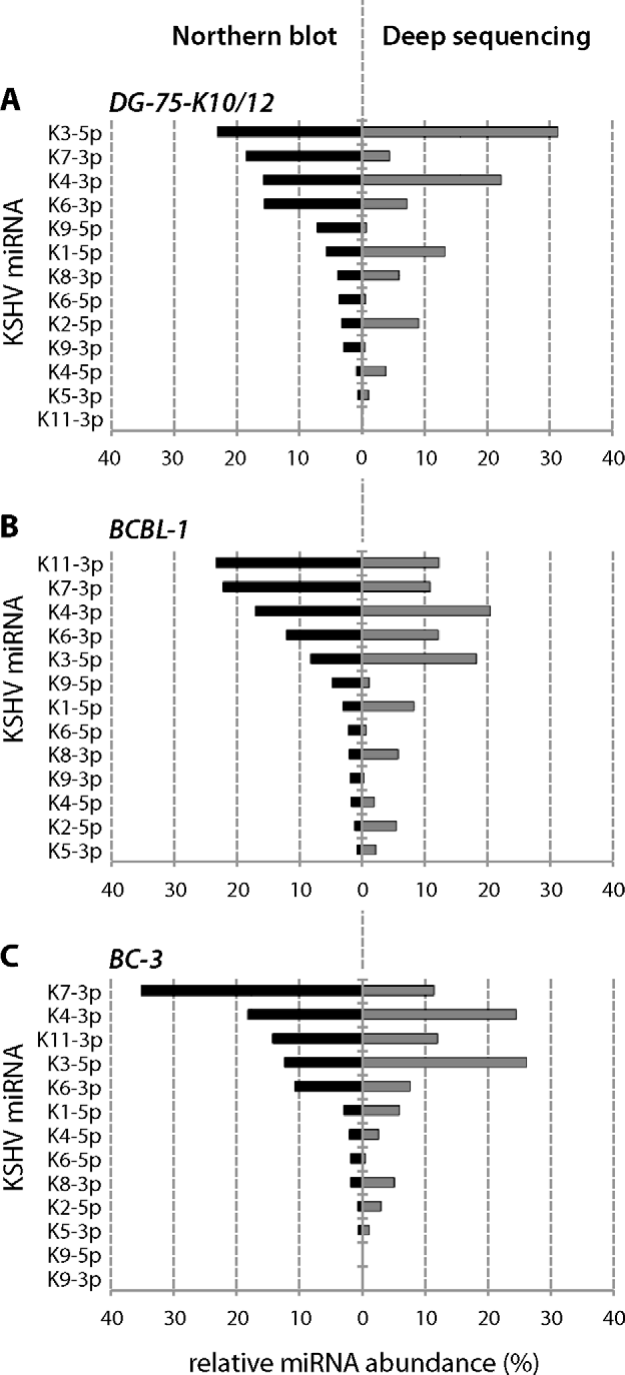
Relative miRNA abundance. KSHV miRNA abundance is given in percentage and was determined by using the total number of fmol (northern blot) or of reads (deep sequencing) of mature miRNAs as 100%. For each cell line, the miRNAs are ranked from the most to the least expressed according to northern blot analyses.

**Figure 3.**
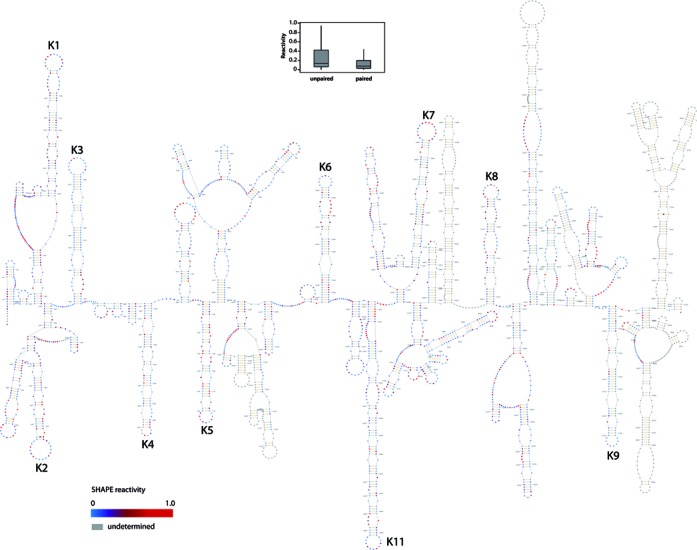
Secondary structure of KSHV pri-miR-K10/12. The secondary structure of pri-miR-K10/12, containing the intronic clustered pre-miRNAs (from pre-miR-K1 to -K9 and pre-miR-K11, as indicated in black), was determined in solution by SHAPE method. The 3157 nt-long RNA we used for the analysis was *in vitro* transcribed. Residues are colored with a gradient of blue to red according to the intensity of SHAPE reactivity (low to high, respectively). Nucleotides in gray were not determined. Box plots represent the SHAPE reactivity in function of the unpaired or paired fate of nucleotides in the 2D structure model. Paired nucleotides show a less scattered pattern than the one for unpaired nucleotides. Moreover median is smaller for paired than unpaired nucleotide.

**Figure 4.**
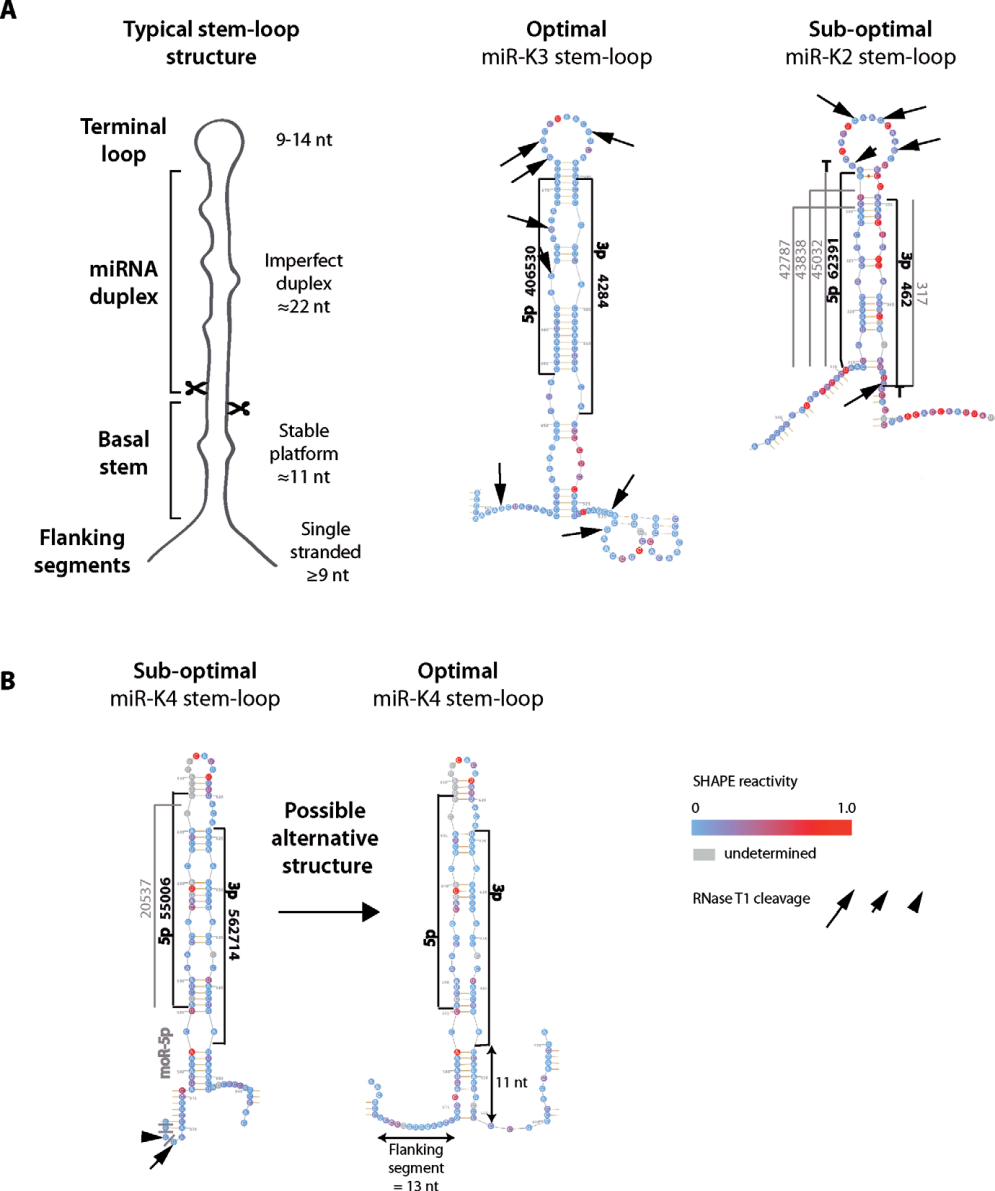
Secondary structure of typical and representative KSHV miRNA stem-loops. (**A**) On the left is depicted a typical miRNA stem-loop structure, with the different modules emphasized (see main text for details). miR-K3 and miR-K2 stem-loops (respectively optimally and suboptimally structured), composed of the pre-miRNA plus 20 nucleotides before and after the Drosha cleavage site, are represented as examples of optimal and suboptimal hairpin structures. SHAPE reactivity of each nucleotide is indicated. Arrows whose sizes are proportional to enzymatic activity indicate RNase T1 cleavages. The most abundant sequences of 5p and 3p arms of the miRNAs and the different isoforms are indicated in black and in gray, respectively, along with the number of sequence reads obtained by deep sequencing. Non-templated nucleotides found at the ends of the miRNA are shown. (**B**) In the case of miR-K4 stem-loop, manual folding allowed for an optimal alternative structure.

**Figure 5.**
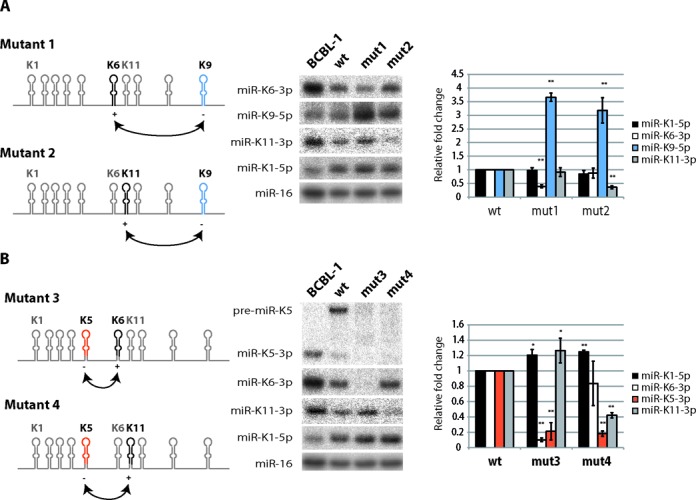
Swapping of KSHV miRNA stem-loops or pre-miRNAs. Either miR-K9 (**A**) or miR-K5 (**B**) stem-loops were swapped with miR-K6 or miR-K11 stem-loops. Left panel: schematic view of the mutants. The miRNA stem-loops (mutants 1 and 2) or the pre-miRNAs (mutants 3 and 4) are indicated in black and in color. The abundance of the corresponding mature miRNAs is indicated by + or −. Middle panel: northern blot analysis of the accumulation of mature miRNAs, after overexpression of wt and mutant plasmids in HEK293Grip cells (*n* = 4). miR-K1–5p and cellular miR-16 were probed as a control of expression and as a loading control, respectively. Right panel: histograms showing the relative expression of the different miRNAs from the mutated plasmids compared to the wt. *P < 0.05 and **P < 0.005.

**Figure 6.**
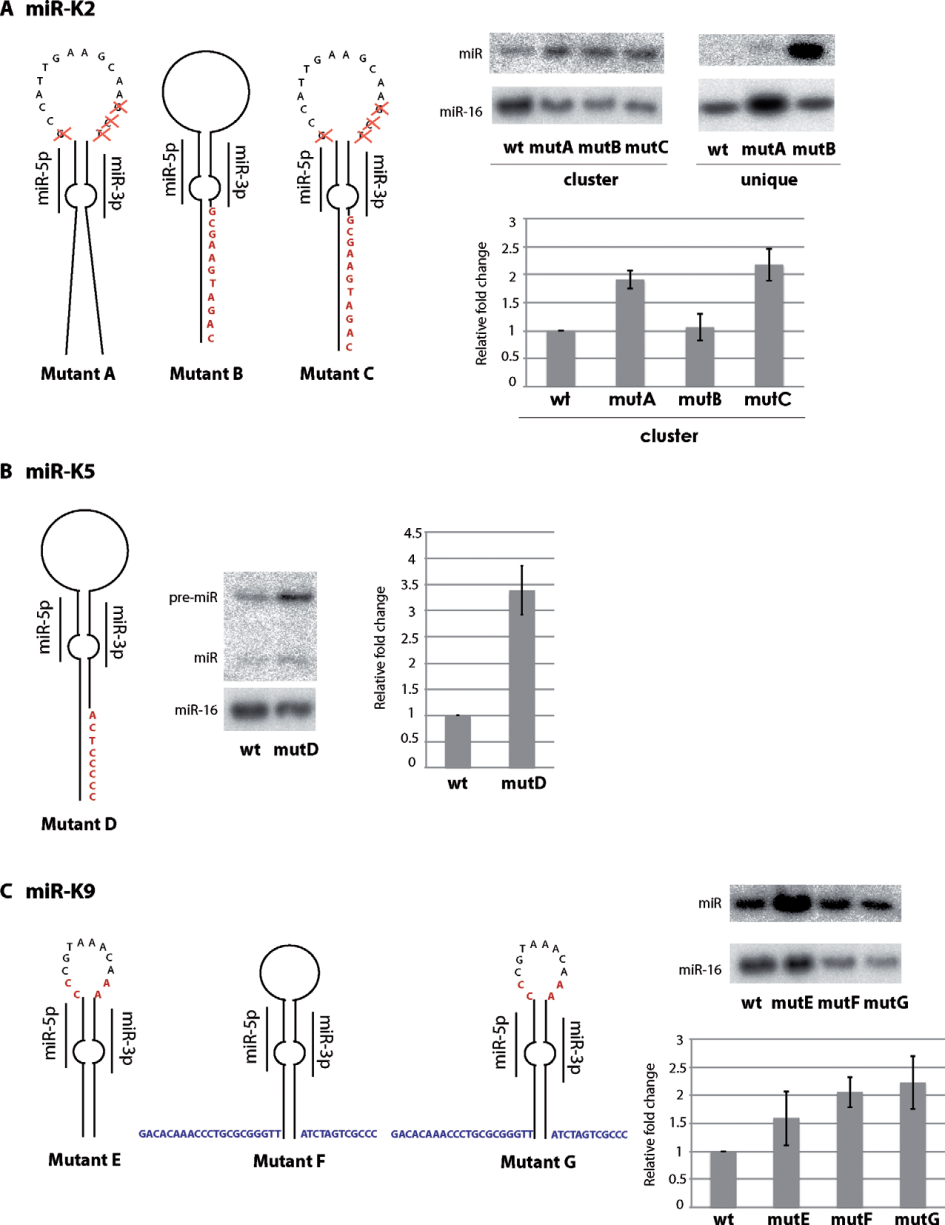
Targeted mutagenesis of suboptimal miRNA stem-loops. miR-K2 (**A**), miR-K5 (**B**) and miR-K9 (**C**) were mutated to optimize the secondary structure. Each missing criterion was added or changed, and the resulting mutants were cloned in the pcDNA-K10/12 or pcDNA-K2 expression plasmid. The resulting constructs were then transfected in HEK293Grip cells and total RNA was analyzed by northern blot for the studied miRNA. miR-16 was probed as a loading control. The histograms represent the relative expression of the different miRNAs from the mutated plasmids compared to the wt for three independent experiments. Inserted nucleotides are indicated in red and nucleotides derived from the miR-K6 stem-loop are indicated in blue, red crosses indicate deletions.

## RESULTS

### Small RNAs expressed in KSHV infected or stable B cell lines

To get the precise sequence of KSHV mature miRNAs, as well as their isoforms, we deep sequenced small RNA libraries (19–33 nt long) deriving from a stable B-cell line expressing the 10 intronic pre-miRNAs (DG-75-K10/12, see Supplementary Figure S1A) (20,21), and two KSHV latently infected primary effusion lymphoma cell lines (BCBL-1 and BC-3). After preprocessing, a total of ∼59.9, ∼52.2 and ∼59.1 M sequences were obtained from the DG-75-K10/12, BCBL-1 and BC-3, respectively (Supplementary Table S2). All three libraries were of good quality (less than 3.2% of ribosomal RNA fragments) and were highly enriched in miRNAs (from ∼57% to ∼72%, Supplementary Figure S2). Viral miRNAs represented ∼9% of the total reads in DG-75-K10/12, BCBL-1 and ∼44% in BC-3.

The sequences of significantly expressed KSHV miRNAs are listed in Supplementary Table S3. Consistent with previous reports, we were able to detect viral miRNAs from both arms in the KSHV infected B-cell lines BCBL-1 and BC-3, apart from miR-K9–5p and -3p in BC-3 (6,38,39). A significant number of reads corresponding to the viral miRNAs, with the notable exception of miR-K11–3p and -5p, was also retrieved from the stable B-cell line DG-75-K10/12 library.

As shown in Supplementary Table S3, the full length and unaltered version of each miRNA did not represent the entire population and sometimes not even the majority of the sequences (see Fraction in %). For example, we observed a non-templated U addition at the 3′ end of miR-K2–3p, shifted 5′ and/or 3′ ends for miR-K1–5p, -K5–5p, -K11–5p, -K7–5p and -3p, -K8–3p, -K10a-3p and -K12–5p, suggesting shifted sites of cleavage by Drosha or Dicer. Mutated versions of miR-K4–5p (C9>U9, 99.75% of the total corresponding miRNA sequences) and miR-K4–3p (A5>G5, 69.21%) were observed in DG-75-K10/12. Given the high proportion of reads corresponding to the mutated miRNA, we can exclude sequencing errors. The same mutations were also present in the miRNA sequences from BCBL-1 and BC-3 cell lines but at very low frequencies (0.13% and 0.02% for C>U in miR-K4–5p in BCBL-1 and BC-3, respectively, and 4.35% and 1.76% for A>G in miR-K4–3p in BCBL-1 and BC-3, respectively). Notably, the corresponding genomic DNA did not contain these mutations, suggesting that the RNAs were edited in the DG-75 cells and with a lower rate in the two infected cell lines (Supplementary Figure S3).

For each KSHV miRNAs, we analyzed the sequences of the different isoforms. To allow for more miRNAs to be detected, the mapping stringency was reduced (see Materials and Methods). All the isoforms are listed in Supplementary Table S4. Altogether, sequences of miRNA isoforms reflect the biogenesis of the corresponding miRNAs, i.e. sequence heterogeneity may arise from imprecise cropping or dicing of the precursor, post-transcriptional modifications (terminal trimming or addition of non-templated nucleotides, modifications). We also observed moRs and AS-miRNA sequences for some KSHV miRNAs that were detectable by northern blot analysis (Supplementary Figure S4). These were mostly the same as those described previously (6). MoR sequences mapped just upstream of the pre-miRNAs and their 3′ end coincided with the 5′ end of the following miRNA sequence (Supplementary Figure S5). The 5′ end of the moRs was less clearly defined.

### Clustered miRNAs from KSHV accumulate at various levels in B-cells

Dramatic differences were observed between the read frequencies of each viral miRNAs (Supplementary Table S3), indicating that they accumulate to different levels in the cell. To validate this observation, we turned to a quantitative northern blot approach to evaluate the absolute amount of KSHV mature miRNAs in DG-75-K10/12, BCBL-1 and BC-3 cells. We restricted this analysis to the mature strands of the clustered miRNAs, with the addition of miR-K4–5p, -K6–5p and -K9–5p that appear to be significantly expressed in our libraries. mir-K9–5p is referenced in miRBase as the passenger arm, but may be more expressed than the 3p arm according to our deep sequencing data.

We detected almost all tested miRNAs in the three cell lines, with the exception of miR-K9–5p and -3p, and miR-K11–3p that were absent in BC-3 and in DG-75-K10/12 cells, respectively. For some miRNAs, additional bands were observed, either above or below the full-length miRNA, as indicated on the blot by -1, -2 or +1 bands (Figure 1A). For miR-K1–5p, miR-K3–5p and miR-K4–3p, an asterisk indicates small RNA species that could correspond to 3′-truncated miRNAs, similar to the previously reported 17-nt long miR-K1–5p (40). For some miRNAs, we could also detect the precursor sequence, although not in a reproducible manner.

Absolute amounts of mature miRNAs were quantified and are compiled in Table 1. Interestingly, BC-3 global expression of viral miRNAs is ∼3.8-fold higher than in BCBL-1 (∼14174 versus ∼3769 10-3 fmol/µg of total RNA). This confirms deep sequencing data from other groups (38,39) and our results where viral miRNA sequences in BC-3 represent up to 44% of total small RNAs versus 9% in BCBL-1. This higher expression of viral miRNAs may correlate with a ∼3-fold increase in the number of KSHV episomes per cell in BC-3 compared to BCBL-1 cells (41). The lower expression of viral miRNAs in DG-75-K10/12 is probably due to the expression of the transgene from a cytomegalovirus (CMV) promoter.

**Table 1.** Absolute quantification of KSHV miRNAs from various cell lines. miRNA accumulation is defined by an absolute amount (fmol of miRNA per µg of total RNA) and by a relative percentage of expression.

	DG-75-K10/12		BCBL-1		BC-3	
	10^−3^ fmol/μg	%	10^−3^ fmol/μg	%	10^−3^ fmol/μg	%
miR-K1–5p	96.28 ± 3.79	5.66	114.33 ± 8.16	3.03	407.48 ± 35.66	2.87
miR-K2–5p	53.58 ± 10.26	3.15	43.25 ± 2.83	1.15	90.59 ± 13.23	0.64
miR-K3–5p	389.36 ±98.73	22.89	307.11 ± 80.96	8.15	1739.90 ± 394.52	12.28
miR-K4–5p	14.28 ± 3.27	0.84	64.48 ± 6.38	1.71	278.06 ± 45.74	1.96
miR-K4–3p	266.03 ± 30.92	15.64	638.33 ± 94.78	16.93	2577.42 ± 477.58	18.18
miR-K5–3p	10.68 ± 3.99	0.63	30.29 ± 7.94	0.80	78.12 ± 13.06	0.55
miR-K6–5p	60.26 ± 21.36	3.54	85.22 ± 13.17	2.26	257.43 ± 68.29	1.82
miR-K6–3p	263.15 ± 44.71	15.47	451.99 ± 41.72	11.99	1509.03 ± 279.25	10.65
miR-K11–3p	-	0.00	872.40 ± 162.05	23.14	2003.58 ± 598.64	14.14
miR-K7–3p	312.61 ± 146.44	18.38	830.43 ± 219.40	22.03	4978.93 ± 1115.48	35.13
miR-K8–3p	64.61 ± 50.35	3.80	79.12 ± 19.28	2.10	253.70 ± 118.76	1.79
miR-K9–5p	121.06 ± 23.94	7.12	181.12 ± 53.32	4.81	-	0.00
miR-K9–3p	49.30 ± 14.01	2.90	71.24 ± 10.72	1.89	-	0.00
Total miRNA	1701.20	100.00	3769.31	100.00	14174.24	100.00

As expected from the deep sequencing data, we observed dramatic differences in the accumulation of viral miRNAs between the three cell lines. We could clearly classify them in two groups based on their level of expression in percentage of all viral miRNAs. Indeed, while miR-K3–5p, -K4–3p, -K6–3p, -K7–3p and -K11–3p (with the exception of DG-75-K10/12 cells where it is absent) accumulate at levels ranging from ∼8% to ∼35%, the other miRNAs are expressed at lower levels (≤7%) (Table 1). The ranking in abundance varies between the different cell lines and may illustrate the importance of the cellular context for miRNA expression. To further explore the contribution of the genomic context for the relative accumulation of each miRNA, we also compared their expression level in transiently transfected HEK293Grip cells using constructs containing either the K10/12 cluster or a portion of it containing only one single pre-miRNA. As shown in Figure 1B, this resulted in notable differences in mature miRNA expression ranging from a complete loss of expression (miR-K2) to a more than 2-fold up-regulation (miR-K8).

Two major differences were observed in the KSHV miRNA profiles of the three cell lines: the absence of miR-K9–5p and -3p in BC-3 and of miR-K11–3p in DG-75-K10/12. As previously reported (38), sequencing of the viral genomic DNA identified several mutations within the pre-miR-K9 sequence in BC-3 compared to the sequence from RefSeq database (NC_009333.1) or to the BCBL-1 genomic sequence (Supplementary Figure S3). Whether these mutations may alter Drosha processing is currently not known. The DG-75-K10/12 cell line was generated by integrating in its genome the 10 intronic clustered miRNAs from BCBL-1 cells. We therefore expected to observe the same viral miRNA expression profile in the two cell lines. However, miR-K11–3p, which is one of the most abundant miRNAs in BCBL-1, does not accumulate in the DG-75-K10/12 cells. We verified that no mutation occurred at the genomic DNA level in the pre-miR-K11 region (Supplementary Figure S3). Surprisingly, this lack of expression seems to be specific of the DG-75 cell line since miR-K11–3p significantly accumulated in the FLP-K10/12 HEK293 derived cell line ((21) and data not shown).

Finally, we compared our deep sequencing and quantitative northern blot data (Figure 2). Globally, miRNAs presenting with the highest number of reads seem to be the most abundant, as assessed by northern blot, but some discrepancies remain between the two technical approaches. This said, the protocol we used to prepare the libraries (with degenerate 3′ and 5′ adapters, (24)) significantly reduced the ligation bias observed in libraries prepared previously using classical adapters (data not shown).

### SHAPE analysis of the KSHV miRNA cluster reveals multiple hairpin structures

In order to determine whether structural features of the primary transcript containing the 10 intronic clustered miRNAs from KSHV (i.e. pri-miR-K10/12) were involved in the differential accumulation of KSHV mature miRNAs, we used hSHAPE (18). This method is very powerful to assess the secondary structure of long RNA, at every single nucleotide. First, the ∼3.2 kb *in vitro* transcribed pri-miR-K10/12 was treated with the electrophilic reagent BzCN that selectively reacts with the 2′-OH of riboses which are conformationally flexible. SHAPE reactivity thus reports on local nucleotide flexibility, i.e. unpaired nucleotides are more reactive.

Figure 3 shows the secondary structure of pri-miR-K10/12; 83% of the 2812 nucleotides analyzed from the 3157 nt of the RNA were resolved, with the exception of some regions that were difficult to retrotranscribe or not interrogated (i.e. the very 3′ extremity). SHAPE constraints were used to create the secondary structure using the RNAstructure (36) and Assemble 2 programs (37). The secondary structure of miRNA-containing regions was additionally confirmed by RNase T1 treatment, a classical enzymatic probe used to assess unpaired G bases (Figure 4 and Supplementary Figure S5). In general, both SHAPE and RNase T1 reactivity correlated well with the single or double-stranded state of nucleotides, i.e. moderately and highly reactive nucleotides are often found in bulges or terminal loops and weakly reactive nucleotides are in helices (Figure 3). Globally, pri-miR-K10/12 is highly structured, folded into multiple hairpin structures (∼30 in total) of various sizes (ranging from 7 to more than 50 bp; smaller hairpins were not considered). Notably, all the miRNA sequences were found in stem-loops, with the 5p and 3p arms forming imperfect duplexes. Many additional hairpin structures were found in pri-miR-K10/12. However, these were not processed, as we did not retrieve small RNA reads from these loci in the deep sequencing data set (not shown).

Interestingly, some regions predicted to be single-stranded were poorly reactive upon SHAPE treatment, suggesting potential long distance tertiary contacts between these regions and other parts of the RNA. For example, pre-miR-K3 and pre-miR-K9, which are moderately reactive to BzCN, show stretches of adenosines in their terminal loops that could be involved in tertiary contacts or stacked with neighboring bases (42). Other pre-miRNAs do not present such features and their nucleotides in the loop are clearly more modified. Alternatively, these regions may be embedded in the tertiary structure and thus not accessible for SHAPE reagent (19). To explore this possibility, pri-miR-K10/12 was treated with moderate and high concentrations of RNase T1. Nevertheless, we could not detect any nuclease resistant product that would correspond to potential embedded regions (data not shown).

### Structural features of KSHV miRNA stem-loops

Recognition and efficient processing of a pri-miRNA by Drosha/DGCR8 relies on specific secondary features. Mutational studies from independent groups have helped to define such criteria (12–14,43,44). Thus, an optimal pri-miRNA is composed of a stem structure of about 3 helical turns (∼33–35 nt long) containing the miRNA duplex at its apical part, terminated by a flexible loop (≥10 nt) and flanked by single-stranded segments at its basal stem (≥9 nt long) (Figure 4A, left panel). The miRNA duplex (∼22 nt) contains numerous mismatches, whereas the basal stem (∼11 nt) is mainly perfectly base-paired, especially at its extremities forming stable platforms (12,13,43,44). In addition, it was described that a UG motif at position −14 and −13 from the 5′ cleavage site, respectively, allowed for the right positioning of Drosha/DGCR8 (44). This basal stem together with flanking single-stranded regions form a key junction for Drosha/DGCR8 recognition (12,13,44). Finally, another key feature is the terminal loop: it should be flexible but not too large since pri-miRNAs with shorter loop or formed by ≥15 nt were shown to be less efficiently processed (14,43). Additionally, a large loop may mimic, along with the miRNA duplex, the junction recognized by Drosha/DGCR8 and may lead to abortive cleavage ∼11 nt from this position (12).

We monitored the presence or absence of each specific criterion, which allowed us to separate the different stem-loops in two groups. Whereas miR-K3, -K6, -K11, -K7 and -K1 stem-loops present optimal structures (containing three to five out of five determinant), miR-K8, -K9, -K5, -K4 and -K2 stem-loops fold into suboptimal stem-loops (containing less than three determinants) (Table 2, Figure 4A and Supplementary Figure S5).

**Table 2.** Structural features of miRNA stem-loops from KSHV pri-miR-K10/12

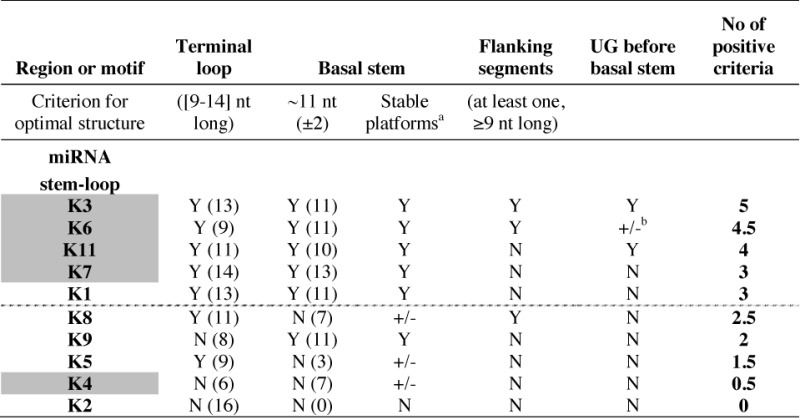						

For each KSHV miRNA stem-loop, the absence (N) or presence (Y) of positive structural criteria for Drosha/DGCR8 recognition and cleavage were determined. miRNA stem-loops were ranked in decreasing order of present determinant, defining two main groups (optimal and suboptimal) separated by a dotted line. Numbers in brackets indicate the length of the terminal loop (in nt) and of the basal stem (in bp). Stem-loops giving rise to abundant mature miRNAs in infected BCBL-1 cells are highlighted in gray.

^a^The basal stem can be paired at one (+/−, value of 0.5) or both extremities (Y), forming stable platforms.

^b^Only presence of a U at position -14 (+/−, value of 0.5).

All the ‘optimal’ miRNA stem-loops possess flexible terminal loops and correct basal stems. The flanking segments were globally less structured than the basal stem. miR-K3, -K6 and -K8 stem-loops present at least one long single-stranded segment (either 5′ or 3′ of the cleavage site). Flanking regions of miR-K11, -K7 and -K1 stem-loops are composed of small hairpins (2–3 bp) surrounded by single-stranded segments. These regions may anyway contribute to the formation of the double-stranded–single-stranded junction important for Drosha/DGCR8 recognition.

The secondary structures of the other KSHV miRNA stem-loops presented with none or only one of the three key features, rendering them suboptimal for Drosha cleavage. Interestingly, none of the other stem-loops present in the sequence and which do not give rise to miRNAs had any of the structural determinants discussed above (data not shown).

### The secondary structural features of miRNA stem-loops correlate with the cellular abundance of mature miRNAs

We compared the secondary structure data to the mature miRNA accumulation in the BCBL-1 cells (Tables 2 and 3). We generally observed a good correlation between the two data sets, with optimal hairpin structures leading to abundant mature miRNAs. However, there are some exceptions, such as miR-K1 and -K4 stem-loops. In the case of miR-K4 stem-loop, we could manually fold it in an alternative, more optimal, structure (Figure 4B). It presents a perfect basal stem of 11 nt with stable platforms and a G at −13 position, flanked by single-stranded segments. Thus, the new structure presents with three out of five determinants, rendering it optimal for Drosha/DGCR8 recognition and cleavage. In the case of miR-K1 stem-loop, another step after Drosha processing, like, for example, the stability, may affect the mature miRNA abundance.

**Table 3.** Correlation between secondary structure, cellular abundance and targeting efficiency of KSHV miRNAs

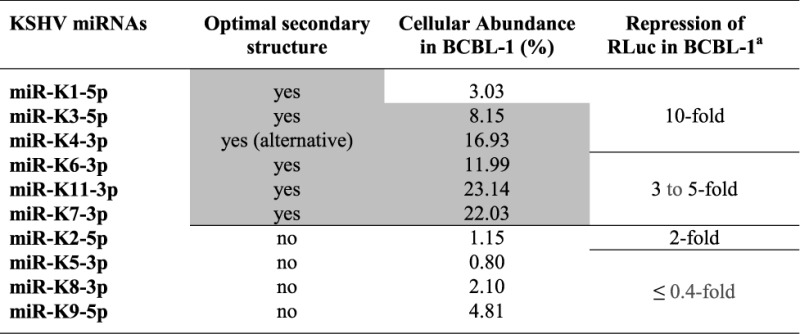			

The miRNAs are ranked according to their capacity to regulate the corresponding RLuc-based indicator constructs harboring two perfectly complementary target sequences into the 3′UTR of the *Renilla* luciferase gene. In gray are emphasized the miRNAs presenting optimal secondary hairpin structures and high levels of abundance in BCBL-1 cells.

^a^From Gottwein *et al.* (46).

Altogether, we conclude that the secondary structure of the miRNA stem-loop controls to a significant level the net accumulation of the corresponding mature miRNA. We suppose that this is mainly due to the impact of structural features on Drosha/DGCR8 recognition and cleavage. Additionally, an optimal stem-loop structure may also be recognized by cellular cofactors that may increase Drosha/DGCR8 and/or Dicer activity and thus contribute to the mature miRNA accumulation.

### Targeted mutagenesis of miRNA stem-loops within pri-miR-K10/12 affects miRNA cellular abundance

In order to test the impact of the secondary structure and the structural context of miRNA expression, we generated various mutant constructs in which we either exchanged the position of individual miRNA stem loops or performed selected point mutations or deletions to optimize the putative secondary structure of the stem-loop. These constructs were then expressed from the pcDNA5 plasmid in human HEK293Grip cells and miRNA expression was quantified by northern blot analysis.

The first set of mutants consisted in swapping whole stem loops and the results are shown in Figure 5. The miR-K9 stem-loop (comprising the entire hairpin structure but not the flanking single-stranded segments) was either swapped with miR-K6 (mutant 1) or miR-K11 (mutant 2) corresponding hairpins. miR-K9–5p is poorly expressed in infected cells compared to miR-K6–3p and miR-K11–3p. These could be due to a suboptimal single-stranded–double-stranded junction or a poor accessibility of the stem-loop structure inside the tridimensional pri-miR-K10/12 (suggested by the weak reactivity of the RNA in this region). In both mutants, the expression of miR-K9–5p was significantly increased (∼3.7-fold for mutant 1 and ∼3.2-fold for mutant 2), whereas those of miR-K6–3p and miR-K11–3p were decreased by ∼2.6- and ∼2.7-fold, in the respective mutants (Figure 5A). This strongly supports our initial hypothesis that swapping of miRNA stem-loops might make a non-optimal structure optimal and vice versa, suggesting that positional context may have an impact.

The two other mutants concerned the swapping of pre-miR-K5 with pre-miR-K6 (mutant 3) or pre-miR-K11 (mutant 4) (Figure 5B). In both cases, the expression of pre-miR-K5 and the corresponding mature miR-K5–3p was unexpectedly strongly decreased (∼4.8-fold for mutant 3 and ∼5.6-fold for mutant 4). This may be due to several reasons. First, the secondary structure of the swapped pre-miR-K5 may be significantly altered and prevent Drosha/DGCR8 recognition and cleavage. Alternatively, the excision of pre-miR-K5 may be highly context dependent and requires additional primary sequence determinants. Finally, these effects might result from a more global change of the pri-miR-K10/12 structure. However, we do not favor this hypothesis since we could observe only minor changes of non-mutated miRNA expression (miR-K1–5p for both mutants, miR-K11–3p for mutant 3 and miR-K6–3p for mutant 4, from ∼1.2- to ∼1.3-fold variation). In addition, the expression of mutated miR-K6–3p and miR-K11–3p showed the expected phenotype. Their accumulation levels were both significantly decreased by ∼9.7- and ∼2.4-fold, respectively, as they were put in place of a poorly expressed miRNA.

The second set of mutants we generated consisted in more precise modifications to insert the missing features in suboptimal stem loops. Thus, we reduced the size of the loop of miR-K2 (mutA), created a basal stem (mutB) or combined the two mutations (mutC). As can be seen in Figure 6A, the shortening of the loop region resulted in a better expression of miR-K2 both in the context of the full cluster and to some extent when miR-K2 was expressed alone. The creation of a basal stem only modestly increase the expression of this miRNA in the context of the cluster, but resulted in a strong overexpression in the context of a unique pre-miRNA construct. Finally, the combination of the two modifications did not impact positively the expression of miR-K2 compared to the loop mutant. Interestingly, the insertion of the basal stem from another miRNA (miR-K6) or of the basal stem and the flanking segments of the same miRNA resulted in a drop in the accumulation of miR-K2 (Supplementary Figure S6A), something we also observed for miR-K5 (Supplementary Figure S6B). These last results are consistent with the observations made in Figure 5B, where the insertion of the miR-K5 stem-loop in the context of miR-K6 resulted in a complete loss of expression. We could also optimize the expression of miR-K5 by creating a basal stem (Figure 6B), and of miR-K9 by increasing the size of the loop or by inserting flanking segments (Figure 6C). This latter result again confirmed the observation made using the swapping mutants where the K9 stem-loop was inserted in the genomic location of the K6 stem-loop.

Altogether, these results indicate that structural features and positional context of miRNA stem-loops into a long pri-miRNA significantly affect mature miRNA accumulation in cells.

## DISCUSSION

It is well established that miRNA biogenesis is a highly regulated process. Indeed, miRNA expression is programmed in space and time, and aberrant accumulation or absence of a given miRNA may lead to diseases. Here, we looked into the importance of the secondary structure of a long viral pri-miRNA containing 10 miRNA precursors for the accumulation of mature miRNAs. Using deep sequencing and quantitative northern blot analysis, we confirmed that individual KSHV miRNAs accumulate to dramatically different levels. We then turned to the SHAPE approach to determine the secondary structure of the long primary transcript. This allowed us to define processing determinants for each miRNA stem-loop and we showed that miRNAs deriving from poorly structured stem-loop accumulated to lower levels compared to miRNAs originating from optimally structured precursors. Using targeted mutagenesis, we found that the position of a given miRNA stem-loop, and thus the structural context, within the pri-miRNA plays an important role for its expression. Finally, we demonstrated that optimizing the secondary structure of suboptimal miRNA stem-loops, such as miR-K2 or miR-K5, resulted in an increase in their expression.

As previously reported, we showed from our deep sequencing data that miRNAs are not represented in the cells as a unique population of sequences but as a broad variety of miRNA isoforms. They can differ by their 5′ or 3′ ends, show addition of non-templated nucleotides or result from editing. For example, we observed edited versions of miR-K4–5p (C9>U9) and miR-K4–3p (A5>I5). Interestingly, the edited versions of miR-K4–5p and miR-K4–3p were found at very low levels in infected B-cells, whereas they were overrepresented in the stable B-cell line. This suggests that the context of infection may inhibit the editing process of these miRNAs. Regarding nucleotide addition, we observed mono-uridylation of miR-K2–3p. Pre-miR-K2 hairpin structure lacks the 3′ 2-nt overhang, rendering it likely a suboptimal substrate for Dicer. Recently, Heo *et al.* showed that the pre-let-7 family also misses this feature, which significantly affects Dicer processing (45). The cell seems to cope with that by subjecting the defective pre-miRNAs to mono-urydilation, restoring the 3′ 2-nt overhang and increasing Dicer maturation. Therefore, pre-miR-K2 may be modified for the same reason.

In our study, we showed that the cellular abundance of the KSHV miRNAs differs dramatically among themselves, and this was dependent of the cellular context. This might be directly connected to the physiology of the cell and may depend on the necessity to regulate at defined levels corresponding miRNA targets. Indeed, concentrations of RNA molecules have to be precisely defined in order to reach the optimal cellular environment. Gottwein *et al.* previously reported on the mRNA targeting efficiency of the KSHV miRNAs, using luciferase reporter assays (46). We compared these data to our results on the cellular abundance and the structural features of the viral miRNAs in BCBL-1 (Table 3). Our study correlated well with Gottwein's data. It confirms that miRNA targeting efficiency is mainly governed by cellular abundance, an idea that was only speculated by the authors at that time. It would be interesting to identify the relative importance of each KSHV miRNAs in terms of associated diseases.

To understand how KSHV miRNA expression is regulated, we looked into the role of the secondary structure of the primary transcript. Many RNA molecules need to form an extremely precise secondary structure for their functions (e.g. rRNAs, tRNAs, etc.). In the case of miRNAs, the stem-loop structure, formed by distinct and defined modules, is important for the maturation by Drosha. Feng *et al.* showed that structural features determine the efficiency of Drosha processing *in vitro* and this correlates with the *in vivo* accumulation of miRNAs (47). This is in accordance with our data where highly expressed KSHV miRNAs are embedded in optimal secondary structures and poorly expressed miRNAs in suboptimal ones. We further proved the importance of specific criteria, such as the size of the terminal loop, the presence of a basal stem or of single-stranded flanking segments, by generating optimized mutants where we could increase the accumulation of otherwise lowly expressed miRNAs. However, we also observed that in some cases, the displacement out of the natural context of sequences flanking the miRNA stem-loop could have a dominant negative effect. Thus, the basal stem and flanking segments of miR-K6, which is optimally structured and well expressed, appear to abolish the correct processing of other miRNA stem-loops. This may be due, for example, to the duplication of important binding elements within the miRNA cluster or simply to the disruption of a long-distance interaction.

Up to now, only scarce data in the literature report on clustered miRNAs, and these concern exclusively the human miR-17–92a cluster. This cluster is composed of six well-folded stem-loops: miR-17, miR-18a, miR-19a, miR-20a, miR-19b-1 and miR-92a-1. Chaulk *et al.* demonstrated that this cluster is composed of a core domain found in a compact globular tertiary structure and protected from Drosha cleavage. The authors propose a role of the tridimensional structure on the regulation of miR-17–92a expression, where the accessibility of pre-miRNAs determines the efficiency of Drosha processing. This would explain the low expression of miR-92, which is embedded within the core domain (15). Chakraborty *et al.* confirmed the importance of the tertiary structure. These authors made a shuffled pri-miR-17–92a where they switched the two halves of the molecule, resulting in pri-miR-20a-19a. There was a marked increase in the levels of various pre-miRNAs (pre-miR-20a, -19a and -92a), indicating a positional importance of the pre-miRNAs in the cluster (16). Similarly, we showed with mutants that the position of miRNA stem-loops within the pri-miR-K10/12 cluster is critical for the expression of mature miRNAs. We could not conclude regarding the existence of a core domain in the KSHV cluster. However, miR-K3 and -K9 stem-loops may be involved in tertiary interactions via stretches of adenosines in their respective apical loop, rendering them less accessible to Drosha. Moreover, sequences or structural features distinct from miRNA stem-loops may also constitute regulatory elements for Drosha cleavage. Finally, the processing of one miRNA may influence the subsequent processing of adjacent miRNAs.

Our data provide evidence that the secondary structure of the pri-miRNA is important for the good expression of clustered miRNAs. There are, however, limitations to our approach as we assessed the secondary structure of the pri-miR-K10/12 cluster using naked RNA. Indeed, some accessory proteins may act by inducing conformational changes in the RNA to satisfy or prevent the structural requirements for Drosha processing. Our results indicate that one of KSHV miRNA, miR-K4–3p, which is expressed at high levels, is embedded into a suboptimal secondary structure. However, we could predict an alternative hairpin structure that would be optimal for Drosha/DGCR8 recognition and cleavage. This alternative structure was not favored in terms of energy and may therefore require a specific RNA chaperone to be induced. Further work will be needed to assess this question in the particular case of miR-K4 stem-loop. Another limitation is that the Drosha processing step could occur cotranscriptionally (48) and, therefore, that the full-length primary transcript does not have time to accumulate. We do not believe that it is the case here because the miRNA cluster is located within an intron and there is ample evidence that the spliced mRNA accumulates (5), if indeed Drosha was cleaving stem-loops as they are being transcribed, then the intron would not have time to be formed and the pre-mRNA would be degraded by exoribonucleases (49). It is true, however, that the pre-miRNA processing could occur during splicing (50) and the full-length intron is short-lived, but the pri-miRNA must have sufficient time to accumulate and therefore to fold before being matured.

In addition to the importance of secondary structure, the primary sequence might also play a role in controlling the processing of miRNA precursors, as recently assessed by the Bartel laboratory (44). Indeed, some sequence motifs are conserved in human pri-miRNAs and the absence of these elements in sequences from *Caenorhabditis elegans* prevent them from Drosha cleavage in human cells. Hence, a U_-14_G_-13_ motif present at the basal stem of the pri-miRNA, a CNNC motif at 17–18 nt downstream of the Drosha cleavage and UGUG sequence at position P24-P27 in the loop all favor the processing by Drosha. We looked for these specific motifs within the KSHV pri-miRNA. We found that well-expressed miRNAs that are embedded in optimal hairpin structures possess at least one of these determinants. For example, miR-K3 stem-loop that has the most optimal structure has the 3 primary sequence determinants. On the other hand, the least expressed miRNAs, miR-K2, -K5 and -K9, with non-optimal secondary structures, have none of these determinants.

It is worth noting that other viruses express their miRNAs in cluster. Interestingly, this is the case for another gammaherpesvirus, the Epstein–Barr virus (EBV), while betaherpesviruses, such as CMVs, have miRNAs dispersed along their genome. The advantage of expressing miRNAs from one locus is evident as the transcriptional control is much more easy to achieve. For viruses, such as KSHV and EBV, which go rapidly into latency, it therefore makes sense to be able to express all miRNAs at once. However, the disadvantage will be that maybe each individual miRNA is not equally important within the cluster. Therefore, there is a need to modulate the accumulation of each miRNA. This regulation can occur at many different levels, and our results provide a first step to understand how the expression of such clustered miRNAs is regulated.

## SUPPLEMENTARY DATA

Supplementary Data are available at NAR Online.

SUPPLEMENTARY DATA
