# Supplementary material for: Importance of the RNA secondary structure for the relative accumulation of clustered viral microRNAs
Source: Nucleic Acids Res. 2014 May 15;42(12):7981–96. doi: 10.1093/nar/gku424 (PMC4081064; doi:10.1093/nar/gku424)
Supplement: SUPPLEMENTARY DATA [file supp_42_12_7981__index.html]

Importance of the RNA secondary structure for the relative accumulation of clustered viral microRNAs — Importance of the RNA secondary structure for the relative accumulation of clustered viral microRNAs — SUPPLEMENTARY DATA 

# Importance of the RNA secondary structure for the relative accumulation of clustered viral microRNAs

## SUPPLEMENTARY DATA

**Files in this Data Supplement:**

- SUPPLEMENTARY DATA
