# Supplementary material for: Importance of the RNA secondary structure for the relative accumulation of clustered viral microRNAs
Source: Nucleic Acids Res. 2014 May 15;42(12):7981–96. doi: 10.1093/nar/gku424 (PMC4081064; doi:10.1093/nar/gku424)
Supplement: SUPPLEMENTARY DATA [file supp_gku424_nar-03603-a-2013-File008.pdf]

Importance of the RNA secondary structure for the relative accumulation of clustered viral microRNAs

SUPPLEMENTARY DATA

Maud Contrant[#], Aurélie Fender[#]*, Béatrice Chane-Woon-Ming, Ramy Randrianjafy, Valérie Vivet-Boudou, Delphine Richer and Sébastien Pfeffer*

Architecture et Réactivité de l'ARN – UPR 9002, Institut de Biologie Moléculaire et Cellulaire du CNRS, Université de Strasbourg, 15 rue René Descartes, F-67084 Strasbourg Cedex, France.

[#] These authors equally contributed to the work.

* To whom correspondence should be addressed.
E-mail: s.pfeffer@ibmc-cnrs.unistra.fr; a.fender@ibmc-cnrs.unistra.fr
Fax: 33 3 88 60 22 18

Supplementary Material and methods

Determination of KSHV genomic DNA sequence.

After extraction using Tri-reagent (MRC, Inc), 25 ng of genomic DNA (BC-3; BCBL-1; DG-75-K10/12) were used to PCR amplify the KSHV genomic region (119270-121967) in 25 μL containing 1 U of Phusion enzyme (Finzyme), 1x HF Phusion buffer (Finzyme), and 0.5 μM of forward primer 21.119 and reverse primer 24.91 as follows: 30" at 98°C, followed by 35 cycles of 10" at 98°C, 30" at 55°C, 2 min at 72°C and a final 5 min at 72°C. The PCR products were purified on a 1% agarose gel and sequenced by GATC Biotech (France).

Table S1. Sequences of the oligonucleotides used in this study.

Name	Sequence (5' to 3')	Usage
RNA preparation	GAAATTAATACGACTCACTATAGAATGCGTGCTTCTGTTTGAAGG	forward primer
	TTTACCGAAACCACCCAGAGGC	reverse primer
Sequencing of KSHV genomic DNA	TCAGCTAGGCCTCAGTATTCTA	reverse primer
	ACAGAACAATAACGGGCGACTAG	reverse primer
	CAGGATAGCCACCCACAATTG	reverse primer
	GTATTTACATCTACGGACCCCC	reverse primer
	CAATCAGCTTGGGAGTTCTAG	reverse primer
	GAAATTAATACGACTCACTATAGGACCGTGAGCGACTCGAAAAAT	forward primer
Mutagenesis	GCGTCTGGATCGACACAAACCCTGCGCGGGTTTCCCAGCTGGGTCTACCCAGCTGCGTAAACCCCGCTGCGTAAACACAGCTGGG	forward for insertion of pre-K9 module into pre-K6 module frame
	GAAGAACACACAGAACAATAACGGGCGACTAGATCCCAGCCGGGTTTACGCAGCTGCGTATACCCAGCTGTGTTTACGCAGCGGG	reverse for insertion of pre-K9 module into pre-K6 module frame
	GCTGAGTCATCGCAGCCCCTATTCCAGTAGGTATAAGAAAGACTTGTCCAGCAGCACCTAATCCATCGGCGGTGCGGGCTGATG	forward for insertion of pre-K6 module into pre-K9 module frame
	CCTTGTTTAATTGTAGAATTACAGCTGGATTTAAAAAAGACTCGCTCAACAGCCCGAAAACCATCAGCCCGCACCGCCGATGGATTAG	reverse for insertion of pre-K6 module into pre-K9 module frame
	GCTGAGTCATCGCAGCCCCTATTCCAGTAGGTATATAAATCCAGCTGTAATTCTACAATTAAACAAGG	forward for deletion of pre-K9 module
	CCTTGTTTAATTGTAGAATTACAGCTGGATTTATATACCTACTGGAATAGGGGCTGCGATGACTCAGC	reverse for deletion of pre-K9 module
	GCGTCTGGATCGACACAAACCCTGCGCGGGTTTATCTAGTCGCCCGTTATTGTTCTGTGTGTTCTTC	forward for deletion of pre-K6 module
	GAAGAACACACAGAACAATAACGGGCGACTAGATAAACCCGCGCAGGGTTTGTGTCGATCCAGACGC	reverse for deletion of pre-K6 module
	GGAATCCTACCTCCACGCTCGCGTATGCCTCATGCCCAGCTGGGTCTACCCAGCTGCGTAAACCCCGCTGCGTAAACACAGCTGG	forward for insertion of pre-K9 module into pre-K11 module frame

	GGTCGGGAACCCGCCCGCGCTTCCGCAAGGCACCCAGCCGGGTTTACGCAGCTGCGTATACCCAGCTGTGTTTACGCAGCGGGG	reverse for insertion of pre-K9 module into pre-K11 module frame
	GCTGAGTCATCGCAGCCCCTATTCCAGTAGGTATACGAGGTTCGCTTTGGTCACAGCTTAAACATTTCTAGGGCGGTGTTATGATCCTTAATG	forward for insertion of pre-K11 module into pre-K9 module frame
	CCTTGTTTAATTGTAGAATTACAGCTGGATTTACGGGGCCGCATCGGACACAGGCTAAGCATTAAGGATCATAACACCGCCCTAG	reverse for insertion of pre-K11 module into pre-K9 module frame
	GGAATCCTACCTCCACGCTCGCGTATGCCTCATGTGCCTTGCGGAAGCGCGGGCGGGTTCCCGACC	forward for deletion of pre-K11 module
	GGTCGGGAACCCGCCCGCGCTTCCGCAAGGCACATGAGGCATACGCGAGCGTGGAGGTAGGATTCC	reverse for deletion of pre-K11 module
	CGACACAAACCCTGCGCGGGTTTAGAAAGACTTGTTAGGTAGTCCCTAGTGCCCTAAGGGTCTACATCAAGCACTTAGG	forward for insertion of pre-K5 into pre-K6 frame
	CAGAACAATAACGGGCGACTAGATAAAAAGACTCGACCGGCAAGTTCCAGGCATCCTAAGTGCTTGATGTAGACCCTTAGG	reverse for insertion of pre-K5 into pre-K6 frame
	GGGCACGGCGTCATGACTAAGGGGGAGTTTGACCCCAGCAGCACCTAATCCATCGGCGGTGCGGGCTGATGG	forward for insertion of pre-K6 into pre-K5 frame
	GTGGCACCAGGTTATGGTCTTATGAGCGGGCTTGCTCAACAGCCCGAAAACCATCAGCCCGCACCGCCGATGGATTAGG	reverse for insertion of pre-K6 into pre-K5 frame
	GGGCACGGCGTCATGACTAAGGGGGAGTTTGACCCAAGCCCGCTCATAAGACCATAACCTGGTGCCAC	forward for deletion of pre-K5
	GTGGCACCAGGTTATGGTCTTATGAGCGGGCTTGGGTCAAACTCCCCCTTAGTCATGACGCCGTGCCC	reverse for deletion of pre-K5
	CGACACAAACCCTGCGCGGGTTTAGAAAGACTTGTCGAGTCTTTTTATCTAGTCGCCCGTTATTGTTCTG	forward for deletion of pre-K6
	CAGAACAATAACGGGCGACTAGATAAAAAGACTCGACAAGTCTTTCTAAACCCGCGCAGGGTTTGTGTCG	reverse for deletion of pre-K6
	CGCTCGCGTATGCCTCATGCGAGGTTCGCTTTTAGGTAGTCCCTAGTGCCCTAAGGGTCTACATCAAGCACTTAGG	forward for insertion of pre-K5 into pre-K11 frame
	CCCGCCCGCGCTTCCGCAAGGCACGGGGCCGCAACCGGCAAGTTCCAGGCATCCTAAGTGCTTGATGTAGACCCTTAGG	reverse for insertion of pre-K5 into pre-K11 frame

	GGGCACGGCGTCATGACTAAGGGGGAGTTTGACCGGTCACAGCTTAAACATTTCTAGGGCGGTGTTATGATCCTTAATGC	forward for insertion of pre-K11 into pre-K5 frame
	GTGGCACCAGGTTATGGTCTTATGAGCGGGCTTGTCGGACACAGGCTAAGCATTAAGGATCATAACACCGCCC	reverse for insertion of pre-K11 into pre-K5 frame
	CGCTCGCGTATGCCTCATGCGAGGTTCGCTTTTGCGGCCCCGTGCCTTGCGGAAGCGCGGGCGGG	forward for deletion of pre-K11
	CCCGCCCGCGCTTCCGCAAGGCACGGGGCCGCAAAAGCGAACCTCGCATGAGGCATACGCGAGCG	reverse for deletion of pre-K11
	CTTCGCTAACTGTAGTCCGGGTCGATCTGACCATTGAAGCAATCCAGATCTTCCAGGGCTAGAGCTGCCGCG	forward mutant A
	CGCGGCAGCTCTAGCCCTGGAAGATCTGGATTGCTTCAATGGTCAGATCGACCCGGACTACAGTTAGCGAAG	reverse mutant A
	CTTCCAGATCTTCCAGGGCTAGAGCTGGCGAAGTAGACCCAATTATAGAAATTTGGTCCCCAAACTCC	forward mutant B
	GGAGTTTGGGGACCAAATTTCTATAATTGGGTCTACTTCGCCAGCTCTAGCCCTGGAAGATCTGGAAG	reverse mutant B
	GCACTTAGGATGCCTGGAACTTGCCGGTCAAACTCCCCCATAAGACCATAACCTGGTGCCACAAAAACC	forward mutant D
	GGTTTTTGTGGCACCAGGTTATGGTCTTATGGGGGAGTTTGACCGGCAAGTTCCAGGCATCCTAAGTGC	reverse mutant D
	GGGTCTACCCAGCTGCGTAAACCCCGCTGCCCGTAAACAAACAGCTGGGTATACGCAGCTGCGTAAACCCG	forward mutant E
	CGGGTTTACGCAGCTGCGTATACCCAGCTGTTTGTTTACGGGCAGCGGGGTTTACGCAGCTGGGTAGACCC	reverse mutant E
	GAGTCATCGCAGCCCCTATTCCAGTAGGTATGACACAAACCCTGCGCGGGTTTGCCAGCTGGGTCTACCCAGCTGCGTAAACCCCGCTGCGTAAACACAG	forward mutant F
	GTTTAATTGTAGAATTACAGCTGGATTTAGGGCGACTAGATGCCAGCCGGGTTTACGCAGCTGCGTATACCCAGCTGTGTTTACGCAGCGGGGTTTAC	reverse mutant F
	GAGTCATCGCAGCCCCTATTCCAGTAGGTATGACACAAACCCTGCGCGGGTTTGCCAGCTGGGTCTACCCAGCTGCGTAAACCCCGCTGCCCGTAAACAAACAG	forward mutant G

	GTTTAATTGTAGAATTACAGCTGGATTTAGGGCGACTAGATGCCAGCCGGGTTTACGCAGCTGCGTATACCCAGCTGTTTGTTTACGGCAGCGGGGTTTAC	reverse mutant G
	AGATTAAGATTATAATGGGTCTACTTCGCTTGGAAAGACTTGAACTGTAGTCCGGGTCGATCTGAGCCATTGAAGCAAGCTTCCAG	forward mutant H
	GGACCAAATTTCTATAATTGGTGTCACCGCGGAAAAAGACTCGCAGCTCTAGCCCTGGAAGATCTGGAAGCTTGCTTCAATGGCTCAG	reverse mutant H
	AGATTAAGATTATAATGGGTCTACTTCGCTTGGAAAGACTTGAACTGTAGTCCGGGTCGATCTGACCATTGAAGCAATCCAG	forward mutant I
	GGACCAAATTTCTATAATTGGTGTCACCGCGGAAAAAGACTCGCAGCTCTAGCCCTGGAAGATCTGGATTGCTTCAATGGTCAGATCG	reverse mutant I
	AGATTAAGATTATAATGGGTCTACTTCGCTGACACAAACCCTGCGCGGGTTTGGAAAGACTTGAACTGTAGTCCGGGTCGATCTGAGCCATTGAAGCAAGCTTCC	forward mutant J
	GGACCAAATTTCTATAATTGGTGTCACCGCGGGGGCGACTAGATAAAAAGACTCGCAGCTCTAGCCCTGGAAGATCTGGAAGCTTGCTTCAATGGCTCAGATC	reverse mutant J
	AGATTAAGATTATAATGGGTCTACTTCGCTGACACAAACCCTGCGCGGGTTTGGAAAGACTTGAACTGTAGTCCGGGTCGATCTGACCATTGAAGCAATCC	forward mutant K
	GGACCAAATTTCTATAATTGGTGTCACCGCGGGGGCGACTAGATAAAAAGACTCGCAGCTCTAGCCCTGGAAGATCTGGATTGCTTCAATGGTCAGATCGAC	reverse mutant K
	CGGCGTCATGACTAAGGGGGAGTTTGACCTTGGAAAGACTTGAGGTAGTCCCTAGTGCCCTAAGGGTCTACATCAAGCACTTAGG	forward mutant L
	CACCAGGTTATGGTCTTATGAGCGGGCTTGAAAAAGACTCGACCGGCAAGTTCCAGGCATCCTAAGTGCTTGATGTAGACCCTTAG	reverse mutant L
	CGGCGTCATGACTAAGGGGGAGTTTGACCTGACACAAACCCTGCGCGGGTTTGGAAAGACTTGAGGTAGTCCCTAGTGCCCTAAGGGTCTACATCAAGCACTTAGG	forward mutant M
	CACCAGGTTATGGTCTTATGAGCGGGCTTGGGGCGACTAGATAAAAAGACTCGACCGGCAAGTTCCAGGCATCCTAAGTGCTTGATGTAGACCCTTAG	reverse mutant M
Northern	GCTTACACCCAGTTTCCTGTAAT	probe for miR-K1-5p

probes		
	CAGATCGACCCGGACTACAGTT	probe for miR-K2-5p
	TCGCTGCCGTCCTCAGAATGTGA	probe for miR-K3-5p
	CCTAGAGTACTGCGGTTTAGCT	probe for miR-K4-5p
	TCAGCTAGGCCTCAGTATTCTA	probe for miR-K4-3p
	CCGGCAAGTTCCAGGCATCCTA	probe for miR-K5-3p
	CCGATGGATTAGGTGCTGCTGG	probe for miR-K6-5p
	CTCAACAGCCCGAAAACCATCA	probe for miR-K6-3p
	AGCGCCAGCAACATGGGATCA	probe for miR-K7-3p
	CGTGCTCTCTCAGTCGCGCCTA	probe for miR-K8-3p
	AGCGGGGTTTACGCAGCTGGGT	probe for miR-K9-5p
	TTACGCAGCTGCGTATACCCAG	probe for miR-K9-3p
	TCGGACACAGGCTAAGCATTAA	probe for miR-K11-3p
	CGCCAATATTTACGTGCTGCTA	probe for miR-16
	CGCCCGCATCCGGCCGTCCTG	probe for moR-K1-5p
	AGTTATACTGCCCGGTTCCA	probe for moR-4-5p (LNA)
	GGTCAAACTCCCCCTTAGT	probe for moR-5-5p
	TCCAGCGACACCCATTTAGT	probe for moR-10-5p
	CTAGCTAAACTGCAGTACTCTA	probe for miR-AS-K4-5p
	TGTACCCAGCTGCGTAAACCCCG	probe for miR-AS-K9-5p
	GCTAGGCGCGACTGAGAGAGC	probe for miR-AS-K8-5p
SHAPE		Coordinates according to the pri-miR-K10/12 *in vitro* transcript (1 to 3157)
N	GACTTTCGCGCCGGGAACAC	247 - 266
M	CACAGGCATTGTAGCTGTTGCG	424 - 444
L	TCAGCTAGGCCTCAGTATTCTA	624 - 645
K	TTGTGGCACCAGGTTATGGTC	813 - 833
J	CCCGCATGGTGTCAAATTAG	1060 - 1079
I	ACAGAACAATAACGGGCGACTAG	1312 - 1334
H	CTCATGGGTATTGGACTTGTAGGC	1563 - 1586
G	CAGGATAGCCACCCACAATTG	1817 - 1837
F	ACGATAACCATACATGGATACACGG	1933 - 1957
E	ATAAAAGGATTAGCGCATGC	2192 - 2211
D	ATTAGCAAATTCAAGATGGCGG	2354 - 2375
C	CAATCAGCTTGGGAGTTCTAG	2550 - 2570
B	TGCCTTATTTGGTAATGCTTCCGG	2834 - 2857

Table S2. Annotation of deep-sequenced small RNA libraries.

2mm max	DG-75-K10/12		BCBL-1		BC-3	
	Read count	Read %	Read count	Read %	Read count	Read %
**total**	60082452	100.00	52392539	100.00	59373221	100.00
**filtered out**	139552	0.23	204917	0.39	224929	0.38
**after preprocessing**	59942900	99.77	52187622	99.61	59148292	99.62
**unmapped**	5528858	9.20	5418623	10.34	4824088	8.13
**hsa miRNAs**	34843255	57.99	24829414	47.39	16285781	27.43
**kshv miRNAs**	5584959	9.30	4881657	9.32	26340822	44.36
**hsa other RNAs**	4257971	7.09	9045232	17.26	6493445	10.94
**unannotated**	9727857	16.19	8012696	15.29	5204156	8.77
**total miRNAs**	40428214	67.29	29711071	56.71	42626603	71.79

Table S3. Major KSHV miRNA-5p and -3p sequences obtained by deep sequencing. For each miRNA, the major sequence obtained by deep sequencing and its fraction (among all the sequences retrieved for the given miRNA) is shown. In bold are indicated the residues in the sequenced RNA species that are different from the sequence reported in miRBase v.19 (www.mirbase.org) and that correspond or not (underlined) to the KSHV genomic sequence.

KSHV miRNAs	Cell lines	Most Abundant Sequence (MAS)	Seq ratio	MAS Fraction
**K1-5p**	DG-75-K10/12	ATTACAGGAAACTGGGTGTAAGC	18064.30	36.87
	BCBL-1	ATTACAGGAAACTGGGTGTAAG-	12639.70	27.34
	BC-3	ATTACAGGAAACTGGGTGTAAGC**T**	32260.60	23.79
**K1-3p (*)**	DG-75-K10/12	GCAGCACCTGTTTCCTGCAACC	6.48	70.99
	BCBL-1	GCAGCACCTGTTTCCTGCAACC	0.54	56.25
	BC-3	GCAGCACCTGTTTCCTGCAACC	16.77	46.85
**K2-5p**	DG-75-K10/12	AACTGTAGTCCGGGTCGATCTG	12303.90	39.22
	BCBL-1	AACTGTAGTCCGGGTCGATCTG	8282.47	25.35
	BC-3	AACTGTAGTCCGGGTCGATCTG	16407.20	31.44
**K2-3p (*)**	DG-75-K10/12	GATCTTCCAGGGCTAGAGCTG**T**	69.70	31.48
	BCBL-1	GATCTTCCAGGGCTAGAGCTG**T**	41.77	37.23
	BC-3	GATCTTCCAGGGCTAGAGCTG**T**	126.48	52.95
**K3-5p**	DG-75-K10/12	TCACATTCTGAGGACGGCAGCGA	42653.80	52.73
	BCBL-1	TCACATTCTGAGGACGGCAGCGA	27750.30	49.30
	BC-3	TCACATTCTGAGGACGGCAGCGA	142456.00	49.16
**K3-3p (*)**	DG-75-K10/12	TCGCGGTCACAGAATGTGACA	521.06	57.20
	BCBL-1	TCGCGGTCACAGAATGTGACA	229.63	62.79
	BC-3	TCGCGGTCACAGAATGTGACA	1165.05	44.90
**K4-5p**	DG-75-K10/12	AGCTAAAC**T**GCAGTACTCTAGG	5160.49	69.04
	BCBL-1	AGCTAAACCGCAGTACTCTAGG	3049.87	60.70
	BC-3	AGCTAAACCGCAGTACTCTAGG	14366.90	70.40
**K4-3p**	DG-75-K10/12	TAGA**G**TACTGAGGCCTAGCTGA	30340.10	57.82
	BCBL-1	TAGAATACTGAGGCCTAGCTGA	31102.10	60.89
	BC-3	TAGAATACTGAGGCCTAGCTGA	133565.00	69.94
**K5-5p (*)**	DG-75-K10/12	**T**AGGTAGTCCCT**A**GTGCCCTAAG-	39.25	37.43
	BCBL-1	**T**AGGTAGTCCCT**A**GTGCCCTAAG-	30.22	33.18
	BC-3	**T**AGGTAGTCCCT**A**GTGCCCTAAG-	90.66	29.16
**K5-3p**	DG-75-K10/12	TAGGATGCCTGGAACTTGCCGGT	1452.17	68.71
	BCBL-1	TAGGATGCCTGGAACTTGCCGGT	3357.38	61.02
	BC-3	TAGGATGCCTGGAACTTGCCGGT	6213.66	67.36
**K6-5p**	DG-75-K10/12	CCAGCAGCACCTAATCCATCGG	769.63	41.88
	BCBL-1	CCAGCAGCACCTAATCCATCGG	1094.09	46.88
	BC-3	CCAGCAGCACCTAATCCATCGG	2791.27	37.84
**K6-3p**	DG-75-K10/12	TGATGGTTTTCGGGCTGTTGAG	9787.47	41.78
	BCBL-1	TGATGGTTTTCGGGCTGTTGAG	18510.50	46.04
	BC-3	TGATGGTTTTCGGGCTGTTGAG	41826.80	51.86
**K11-5p (*)**	DG-75-K10/12	GGTCACAGCTTAAACATTTCTAGG**G**	0.05	50.00
	BCBL-1	GGTCACAGCTTAAACATTTCTAGG	2.29	48.53
	BC-3	GGTCACAGCTTAAACATTTCTAGG	178.33	49.70
**K11-3p**	DG-75-K10/12	TTAATGCTTAGCCTGTGTCCGA	77.81	47.55
	BCBL-1	TTAATGCTTAGCCTGTGTCCGA	18684.50	51.69
	BC-3	TTAATGCTTAGCCTGTGTCCGA	65499.20	63.19
**K7-5p (*)**	DG-75-K10/12	AGCGCCACCGGACGGGGATTTAT--	88.94	31.31
	BCBL-1	**TG**AGCGCCACCGGACGGGGAT------	133.95	21.01
	BC-3	AGCGCCACCGGACGGGGATTTA---	1815.85	25.44

**K7-3p**	DG-75-K10/12	TGATCCCATGTTGCTGGCGC**TCA**	6002.93	35.26
	BCBL-1	TGATCCCATGTTGCTGGCGC**T**	16548.00	50.26
	BC-3	TGATCCCATGTTGCTGGCGC**T**	62372.80	45.93
**K8-5p (*)**	DG-75-K10/12	ACTCCCTCACTAACGCCCCGCT	1081.09	53.65
	BCBL-1	ACTCCCTCACTAACGCCCCGCT	1617.99	47.21
	BC-3	ACTCCCTCACTAACGCCCCGCT	6386.01	64.02
**K8-3p**	DG-75-K10/12	CTAGGCGCGACTGAGAGAGCA**C**	8069.62	38.98
	BCBL-1	CTAGGCGCGACTGAGAGAGCA	8744.79	23.41
	BC-3	CTAGGCGCGACTGAGAGAGCA	27914.30	31.12
**K9-5p (*)**	DG-75-K10/12	ACCCAGCTGCGTAAACCCCGCT	988.91	35.39
	BCBL-1	ACCCAGCTGCGTAAACCCCGCT	1764.43	28.79
	BC-3	A**T**CCAG**-**T**AG**GTA**T**ACCC**A**G**G**T	0.00	0.00
**K9-3p**	DG-75-K10/12	CTGGGTATACGCAGCTGCGTAA	621.40	58.02
	BCBL-1	CTGGGTATACGCAGCTGCGTAA	551.54	35.15
	BC-3	CTGGGTATAC**C**CAGCTGCGTAA	0.00	0.00
**K10a-5p (*)**	DG-75-K10/12	GGCTTGGGGCGATACCACCACT	0.37	80.00
	BCBL-1	GGCTTGGGGCGATACCACCACT	27.90	76.60
	BC-3	GGCTTGGGGCGATACCACCACT	443.96	69.38
**K10a-3p**	DG-75-K10/12	TAGTGTTGTCCCCCCGAGTGG-	20.97	21.93
	BCBL-1	TAGTGTTGTCCCCCCGAGTGG-	7121.24	24.21
	BC-3	TAGTGTTGTCCCCCCGAGTGGC	29644.50	22.32
**K12-5p (*)**	DG-75-K10/12	**TC**AACCAGGCCACCATTCCTCT**---**	2.05	20.48
	BCBL-1	**TC**AACCAGGCCACCATTCCTCT**---**	528.92	19.45
	BC-3	**TC**AACCAGGCCACCATTCCTCT**---**	3374.21	22.66
**K12-3p**	DG-75-K10/12	TGGGGGAGGGTGCCCTGGTTGA	22.61	47.05
	BCBL-1	TGGGGGAGGGTGCCCTGGTTGA	2492.99	43.92
	BC-3	TGGGGGAGGGTGCCCTGGTTGA	29025.00	50.92

Table S4. IsomiRNAs, fraction in % of the different isoforms per miRNA. The different categories correspond to: full length (fl), 3' or 5' trimmed (3trim or 5trim), 3' or 5' tailed (3add or 5add) and every possible combination (5trim3trim, 5trim3add, etc). The miRNAs were either identical (id) or different (iso) to the template sequence.

DG-75-K10/12 (9mm max)

KSHV miRNA	GenomicPosition (Ref:Start:End: Strand)	Total read count	fl_id	fl_iso	3trim_id	3trim_iso	3add_id	3add_iso	5trim_id	5trim_iso	5trim3trim_id	5trim3trim_iso	5trim3add_id	5trim3add_iso	5add_id	5add_iso	5add3trim_id	5add3trim_iso	5add3add_id	5add3add_iso
**miR-K1-5p**	NC:009333.1:122137:122159:-	730361	36.87	7.2	9.97	2.64	32.82	8.77	0.37	0.07	0.12	0.02	0.77	0.21	0.01	0.07	0.01	0.02	0.02	0.07
**miR-K1-3p**	NC:009333.1:122100:122121:-	262	70.99	8.02	14.12	0	0	4.96	0	0	0	0	0	0	0	0	1.91	0	0	0
**miR-K2-5p**	NC:009333.1:121977:121998:-	497463	39.22	1.34	33.65	1.5	5.92	16.83	0.48	0.01	0.33	0.02	0.15	0.22	0.05	0.12	0.03	0.09	0	0.05
**miR-K2-3p**	NC:009333.1:121935:121955:-	2818	16.15	0.67	5.36	0.07	24.45	40.17	0.53	0.39	0	0	1.7	0.82	1.95	0.32	3.05	1.1	0.78	2.48
**miR-K3-5p**	NC:009333.1:121834:121856:-	1724546	52.73	1.22	5.57	0.43	23.86	11.81	0.9	0.03	0.09	0.01	0.46	0.27	0.82	0.29	0.65	0.06	0.49	0.32
**miR-K3-3p**	NC:009333.1:121794:121814:-	21067	57.2	2.64	13.02	1.05	0.2	2.63	0.9	0.1	0.07	0.02	0	0.07	13.64	1	6.56	0.71	0.01	0.16
**miR-K4-5p**	NC:009333.1:121703:121724:-	208645	0.16	69.19	0.05	26.27	0	0.96	0	1.06	0	0.5	0	0.02	0	0.15	0.01	1.65	0	0
**miR-K4-3p**	NC:009333.1:121665:121686:-	1226689	23.46	58.77	3.25	7.23	0.35	3.42	1.1	1.86	0.09	0.16	0.04	0.15	0.01	0.09	0	0.02	0	0.01
**miR-K5-5p**	NC:009333.1:121552:121574:-	1587	1.32	2.65	31.63	1.32	0	0.19	0	0	0	0	0	0	0.88	3.47	54.19	4.35	0	0
**miR-K5-3p**	NC:009333.1:121513:121535:-	58713	68.71	8.35	9.78	1.11	0.12	9.08	1.15	0.1	0.19	0	0	0.31	0.46	0.25	0.28	0.05	0	0.06
**miR-K6-5p**	NC:009333.1:121044:121065:-	31117	41.88	0.75	4.3	0.05	34.46	16.14	0.74	0.02	0.04	0	0.99	0.29	0	0.13	0.04	0	0.02	0.14
**miR-K6-3p**	NC:009333.1:121012:121033:-	395720	41.78	0.9	5.03	0.22	26.68	21.36	1.06	0.04	0.08	0	1.84	0.8	0.04	0.06	0	0	0.02	0.07
**miR-K11-5p**	NC:009333.1:120864:120887:-	2	0	50	0	0	50	0	0	0	0	0	0	0	0	0	0	0	0	0
**miR-K11-3p**	NC:009333.1:120827:120848:-	3146	47.55	15.38	15.77	2.92	9.28	7.31	0.16	0.06	0.06	0	0.6	0.06	0.48	0.16	0.13	0.06	0	0
**miR-K7-5p**	NC:009333.1:120644:120667:-	3596	24.89	3.62	51.64	2.17	0.5	5.17	0	0	0.42	0	0	0.81	0.03	0.03	7.62	3.11	0	0
**miR-K7-3p**	NC:009333.1:120608:120627:-	242706	0.91	0.18	0.71	0.01	69.52	27.17	0	0.01	0	0	0.8	0.22	0	0	0	0	0.04	0.41
**miR-K8-5p**	NC:009333.1:120233:120254:-	43710	53.65	1.81	12.22	1.16	19.49	9.85	0.46	0.03	0.19	0	0.12	0.13	0.34	0.35	0.04	0.06	0.08	0.02
**miR-K8-3p**	NC:009333.1:120194:120214:-	326265	17.78	0.28	10.17	0.76	39.74	21.86	1.23	0.03	0.44	0.02	5.63	1.8	0.01	0.08	0.02	0.04	0.01	0.11

**miR-K9-5p**	NC:009333.1:119586:119607:-	39983	35.39	2.11	22.19	9.39	0.28	19.56	0.45	0.01	0.19	0.05	0	0.25	1.37	1.29	4.72	2.1	0	0.67
**miR-K9-3p**	NC:009333.1:119552:119573:-	25124	58.02	3.37	21.96	0.53	6.09	3.41	3.73	0.05	0.92	0.11	1.05	0.19	0	0.53	0	0.02	0	0.02
**miR-K10a-5p**	NC:009333.1:118116:118137:-	15	80	6.67	0	6.67	0	0	6.67	0	0	0	0	0	0	0	0	0	0	0
**miR-K10a-3p**	NC:009333.1:118076:118097:-	848	20.87	8.25	23.7	5.54	0.12	6.13	0.24	0.12	0.12	0	0	0	9.67	5.31	12.03	6.6	0	1.3
**miR-K12-5p**	NC:009333.1:117835:117857:-	83	0	0	4.82	7.23	0	1.2	1.2	0	0	0	0	0	0	0	69.88	15.66	0	0
**miR-K12-3p**	NC:009333.1:117798:117819:-	914	47.05	9.08	18.16	2.3	0.33	20.46	0.11	0.11	0.22	0	0.66	0.22	0.44	0.33	0.22	0.11	0	0.22

BCBL-1 (9mm max)

KSHV miRNA	GenomicPosition (Ref:Start:End:Strand)	Total read count	fl_id	fl_iso	3trim_id	3trim_iso	3add_id	3add_iso	5trim_id	5trim_iso	5trim3trim_id	5trim3trim_iso	5trim3add_id	5trim3add_iso	5add_id	5add_iso	5add3trim_id	5add3trim_iso	5add3add_id	5add3add_iso
**miR-K1-5p**	NC:009333.1:122137:122159:-	375567	26.34	7.07	34.93	2.13	24.43	3.23	0.35	0.11	0.51	0.03	0.57	0.05	0.01	0.07	0.02	0.06	0.01	0.06
**miR-K1-3p**	NC:009333.1:122100:122121:-	16	56.25	18.75	25	0	0	0	0	0	0	0	0	0	0	0	0	0	0	0
**miR-K2-5p**	NC:009333.1:121977:121998:-	246100	25.35	0.87	44.77	5.4	3.04	19.07	0.31	0.01	0.42	0.07	0.08	0.22	0.03	0.07	0.07	0.18	0	0.04
**miR-K2-3p**	NC:009333.1:121935:121955:-	1241	25.54	0.32	11.76	0.56	7.01	43.03	1.69	0.08	0.56	0	1.13	2.01	0	0.56	3.95	0.16	0	1.61
**miR-K3-5p**	NC:009333.1:121834:121856:-	824554	49.3	2.96	16.87	1.21	16.46	9.09	0.61	0.04	0.22	0.01	0.24	0.14	0.7	0.41	1.05	0.17	0.23	0.3
**miR-K3-3p**	NC:009333.1:121794:121814:-	6823	62.79	3.65	21.4	2.93	0.22	3.15	1.22	0.06	0.41	0.22	0	0	3.06	0.21	0.53	0	0	0.16
**miR-K4-5p**	NC:009333.1:121703:121724:-	90622	60.7	1.4	24.9	0.4	0.36	7.41	0.45	0.01	0.25	0	0	0.03	0.32	0.63	2.62	0.43	0	0.08
**miR-K4-3p**	NC:009333.1:121665:121686:-	924147	60.89	9.57	18.43	1.85	2.25	4.53	1.39	0.16	0.31	0.03	0.11	0.13	0.01	0.24	0.01	0.1	0	0.01
**miR-K5-5p**	NC:009333.1:121552:121574:-	898	0	2.67	16.93	0.56	0	0	0	0	0.22	0	0	0	0	5.46	59.8	14.37	0	0
**miR-K5-3p**	NC:009333.1:121513:121535:-	99759	61.02	4.85	16.8	1.27	0.15	12.96	1.11	0.09	0.24	0.03	0	0.26	0.24	0.24	0.5	0.12	0	0.13
**miR-K6-5p**	NC:009333.1:121044:121065:-	32509	46.88	0.7	11.22	0.13	21.17	17.97	0.49	0.02	0.2	0	0.35	0.24	0.02	0.32	0.06	0.08	0	0.14
**miR-K6-3p**	NC:009333.1:121012:121033:-	550009	46.04	1.8	16.63	0.98	5.61	26.01	1.3	0.06	0.31	0.02	0.41	0.62	0.05	0.05	0.02	0.02	0.01	0.04
**miR-K11-5p**	NC:009333.1:120864:120887:-	68	48.53	0	26.47	0	1.47	23.53	0	0	0	0	0	0	0	0	0	0	0	0
**miR-K11-3p**	NC:009333.1:120827:120848:-	555179	51.69	5.87	20.52	1.69	11.3	7.6	0.42	0.04	0.14	0.02	0.13	0.08	0.2	0.11	0.11	0.03	0.03	0.02
**miR-K7-5p**	NC:009333.1:120644:120667:-	3980	7.59	0.63	34.8	2.46	0	1.06	0.03	0	0.28	0	0	0	0.1	0.23	44.12	8.72	0	0

**miR-K7-3p**	NC:009333.1:120608:120627:-	491698	3.29	1.57	4.23	0.06	69.83	19.61	0.03	0.02	0.02	0	0.67	0.22	0	0.01	0	0.03	0.05	0.38
**miR-K8-5p**	NC:009333.1:120233:120254:-	48076	47.21	2.17	23.95	3.68	15.36	6.22	0.38	0	0.19	0.06	0.19	0.06	0.16	0.09	0.08	0.05	0.08	0.06
**miR-K8-3p**	NC:009333.1:120194:120214:-	259837	23.41	0.57	38.41	3.12	7.99	17.81	1.95	0.05	2.44	0.2	1.71	2.08	0.02	0.06	0.03	0.11	0	0.05
**miR-K9-5p**	NC:009333.1:119586:119607:-	52427	28.79	3.91	34.29	13.28	0.07	10.19	0.17	0.01	0.18	0.04	0	0.06	0.74	1.08	4.2	2.78	0	0.22
**miR-K9-3p**	NC:009333.1:119552:119573:-	16388	35.15	3.69	43.18	2.96	3.47	3.52	4.63	0.35	1.47	0.05	0.7	0.16	0.02	0.23	0.11	0.31	0	0.01
**miR-K10a-5p**	NC:009333.1:118116:118137:-	829	76.6	2.29	10.01	0.72	6.15	2.9	0	0.6	0	0	0	0	0	0.72	0	0	0	0
**miR-K10a-3p**	NC:009333.1:118076:118097:-	211596	15.63	8.85	28.02	9.57	0.03	4.46	0.17	0.12	0.37	0.1	0	0.04	5.55	4.87	14.1	6.34	0.01	1.76
**miR-K12-5p**	NC:009333.1:117835:117857:-	15716	2.94	0.2	11.82	1.21	0.01	5.17	0.29	0	0.11	0.04	0.48	1.08	0.01	0.06	61.52	14.91	0.09	0.07
**miR-K12-3p**	NC:009333.1:117798:117819:-	74075	43.92	9.08	22.91	1.61	0.38	20.29	0.41	0.09	0.16	0.03	0.2	0.1	0.19	0.15	0.23	0.13	0	0.1
**BC-3 (9mm max)**																				
**KSHV miRNA**	**GenomicPosition (Ref:Start:End:Strand)**	**Total read count**	**fl_id**	**fl_iso**	**3trim_id**	**3trim_iso**	**3add_id**	**3add_iso**	**5trim_id**	**5trim_iso**	**5trim3trim_id**	**5trim3trim_iso**	**5trim3add_id**	**5trim3add_iso**	**5add_id**	**5add_iso**	**5add3trim_id**	**5add3trim_iso**	**5add3add_id**	**5add3add_iso**
**miR-K1-5p**	NC:009333.1:122137:122159:-	1375274	21.02	4.64	26.69	1.46	39.42	4.25	0.36	0.09	0.53	0.02	1.2	0.11	0.01	0.04	0.01	0.06	0.02	0.09
**miR-K1-3p**	NC:009333.1:122100:122121:-	715	46.85	4.34	37.62	3.64	0	0.98	1.54	0	3.36	0	0	0	0	0.42	1.12	0.14	0	0
**miR-K2-5p**	NC:009333.1:121977:121998:-	699439	31.44	0.81	41.6	5.13	3.21	16.28	0.34	0.01	0.39	0.05	0.15	0.24	0.03	0.1	0.04	0.14	0	0.03
**miR-K2-3p**	NC:009333.1:121935:121955:-	5392	19.57	1.58	5.51	0.11	4.36	57.88	2.06	0.17	0.13	0	0.2	2.1	2.34	0.15	2.76	0.24	0.02	0.83
**miR-K3-5p**	NC:009333.1:121834:121856:-	6072927	49.16	1.77	9.85	0.71	22.29	11.3	0.76	0.03	0.14	0.01	0.36	0.2	0.93	0.34	0.94	0.1	0.69	0.41
**miR-K3-3p**	NC:009333.1:121794:121814:-	49666	44.9	2.11	21.46	2.25	0.09	1.7	0.88	0.09	0.54	0.09	0	0.05	20.5	1.01	3.7	0.3	0.04	0.29
**miR-K4-5p**	NC:009333.1:121703:121724:-	612461	70.4	1.36	17.26	0.24	0.41	6.3	0.47	0.02	0.22	0.01	0	0.04	0.46	0.64	1.86	0.27	0	0.04
**miR-K4-3p**	NC:009333.1:121665:121686:-	5693876	69.94	4.78	15.84	1.04	2.08	3.92	1.47	0.09	0.29	0.01	0.06	0.08	0.02	0.27	0.01	0.08	0	0.01
**miR-K5-5p**	NC:009333.1:121552:121574:-	3865	0.54	2.61	35.76	1.03	0	0.05	0	0.08	0.78	0.03	0	0	0.54	3.44	48.41	6.6	0	0.13
**miR-K5-3p**	NC:009333.1:121513:121535:-	264889	67.36	5.52	10.36	0.75	0.11	12.82	1.44	0.12	0.2	0	0	0.27	0.33	0.26	0.28	0.08	0	0.08
**miR-K6-5p**	NC:009333.1:121044:121065:-	118992	37.84	0.58	9.83	0.15	31.03	16.95	0.62	0.03	0.16	0	1.72	0.51	0.03	0.18	0.01	0.06	0.05	0.25
**miR-K6-3p**	NC:009333.1:121012:121033:-	1783080	51.86	1.42	12	1.07	6	24.93	1.19	0.05	0.2	0.02	0.41	0.64	0.06	0.07	0.02	0.02	0.01	0.04

**miR-K11-5p**	NC:009333.1:120864:120887:-	7602	49.7	0.97	38.04	0.79	2.18	6.95	0.3	0.01	0.34	0.01	0	0.12	0	0.3	0.18	0	0.08	0.01
**miR-K11-3p**	NC:009333.1:120827:120848:-	2792234	63.19	3.81	16.79	1.24	7.84	5.7	0.53	0.03	0.11	0.01	0.13	0.06	0.29	0.1	0.09	0.02	0.03	0.03
**miR-K7-5p**	NC:009333.1:120644:120667:-	77410	17	1.5	57.41	1.86	0.06	1.56	0.32	0.02	0.56	0.03	0	0.05	0.01	0.04	16.77	2.81	0	0
**miR-K7-3p**	NC:009333.1:120608:120627:-	2658959	2.23	1.21	2.7	0.03	74.59	17.73	0.02	0.01	0.01	0	0.8	0.2	0	0.01	0	0.01	0.06	0.38
**miR-K8-5p**	NC:009333.1:120233:120254:-	272236	64.02	1.96	14.85	2.11	10.53	5.05	0.51	0.01	0.16	0.02	0.08	0.04	0.29	0.15	0.06	0.03	0.06	0.05
**miR-K8-3p**	NC:009333.1:120194:120214:-	1189987	31.12	0.57	22.86	2.05	11.88	21.68	2.85	0.05	1.7	0.14	2.37	2.5	0.02	0.07	0.03	0.06	0.01	0.05
**miR-K9-5p**	NC:009333.1:119586:119607:-	82	30.49	2.44	36.59	14.63	0	13.41	0	0	0	0	0	0	0	0	1.22	1.22	0	0
**miR-K9-3p**	NC:009333.1:119552:119573:-	41	31.71	2.44	46.34	0	4.88	0	0	7.32	0	7.32	0	0	0	0	0	0	0	0
**miR-K10a-5p**	NC:009333.1:118116:118137:-	18926	69.38	2.16	15.33	0.82	9.35	1.72	0.69	0.01	0.04	0.02	0.15	0.02	0.04	0.17	0.01	0.05	0.03	0.03
**miR-K10a-3p**	NC:009333.1:118076:118097:-	1263749	22.32	6.24	22.52	5.84	0.03	4.76	0.33	0.08	0.33	0.08	0	0.08	9.74	5.73	12.99	6.55	0.03	2.34
**miR-K12-5p**	NC:009333.1:117835:117857:-	143843	2.22	0.59	9.14	3.38	0.01	3.38	0.19	0.02	0.22	0.1	0.27	0.47	0.04	0.04	62.97	16.93	0	0.03
**miR-K12-3p**	NC:009333.1:117798:117819:-	1237340	50.92	7.96	20.4	1.67	0.44	15.6	0.38	0.08	0.19	0.01	0.8	0.38	0.31	0.29	0.32	0.16	0	0.09

SUPPLEMENTARY FIGURE LEGENDS

Figure S1. (A) **Cluster of KSHV miRNAs.** The twelve KSHV pre-miRNAs are localized in the latency locus and are indicated by red arrows. Ten of them are clustered in an intron (pre-miR-K1 to -K9 and pre-miR-K11) whereas pre-miR-K10 and -K12 are in the coding region and in the 3'UTR of Kaposin mRNA, respectively. Genomic coordinates of the intronic cluster of pre-miRNAs are 122209 to 119153.

(B) **Positions of the RT primers used for SHAPE analysis.** KSHV pri-miR-K10/12 is schematized with the 10 pre-miRNAs in black boxes. Letters (B to N) correspond to the 5' position of the oligonucleotides used for reverse transcription (see Table S1).

Figure S2. Pie chart representations of the different small RNA categories found in the libraries. Values were derived from Table S2.

Figure S3. Alignments of genomic DNA from KSHV cluster from various cell lines compared to the sequence in RefSeq.

The genomic sequences of the three cell lines used in this study (DG-75-K10/12, BCBL-1 and BC-3) were compared to the reference sequence (NC_009333.1). An asterisk indicates sequence variations, and point mutations or deletions are indicated in red. The 5p and 3p arms of the pre-miRNAs are indicated, with the most abundant forms in dark grey and the less abundant in light grey. Genomic positions are indicated.

Figure S4. KSHV miRNA offset RNA (A) and antisense miRNA (B) detection by northern blot analysis.

40 μg of total RNA from DG-75-K10/12, BCBL-1 and BC-3 cell lines were loaded on a 15% PAGE and submitted to northern blot analysis. Total RNA from DG-75-EGFP, expressing no KSHV RNAs, was used as a negative control. Bands corresponding to moRs and AS-miRNAs are indicated by an asterisk. U6 RNA was probed as a loading control. A ladder is indicated next to the autoradiograms.

Figure S5. Secondary structure of KSHV miRNA stem-loops.

MiRNA stem-loops composed of the pre-miRNA plus ~20 nucleotides before and after the Drosha cleavage site are represented. SHAPE reactivity of each nucleotide is indicated. Arrows correspond to cleavage by RNase T1 (after single stranded G residues). Asterisks

indicate the UG motif at -14 and -13 from the 5' of the cleavage site. The most abundant sequences of the miRNA-5p and -3p and the different isoforms are indicated in black and in grey respectively, along with the number of reads obtained by deep sequencing. Non-template nucleotides found at the ends of the miRNA are shown. For some pre-miRNAs, microRNA offset RNAs (moRs) were sequenced and are indicated in grey. They are localized upstream of the miRNA-5p and present variations at their 5' end (as indicated by short lines in grey) whereas the 3' extremity is invariant.

Figure S6. Additional mutants of sub-optimal miRNA stem-loops.
miR-K2 (A) and miR-K5 (B) were mutated to optimize their secondary structure. The resulting mutants were cloned in the pcDNA-K10/12 expression plasmid. The resulting constructs were then transfected in HEK293Grip cells and total RNA was analyzed by northern blot for the studied miRNA. miR-16 was probed as a loading control. Inserted nucleotides derived from the miR-K6 stem-loop are indicated in blue, red crosses indicate deletions.

Figure S1

A

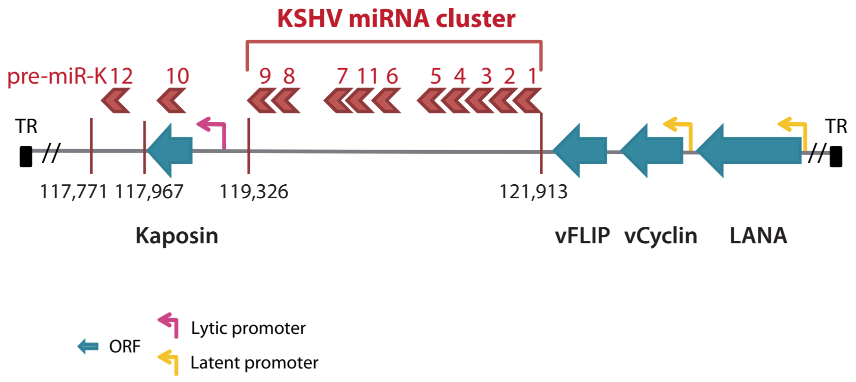

KSHV miRNA cluster
pre-miR-K12
10
9 8
7 11 6
5 4 3 2 1
TR
TR
117,771 117,967
119,326
121,913
Kaposin
vFLIP vCyclin
LANA
ORF
Lytic promoter
Latent promoter

B

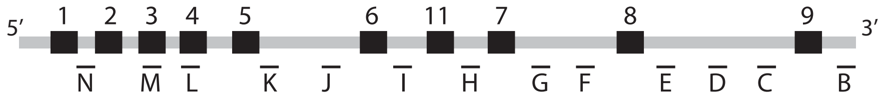

5′
1 2 3 4 5 6 11 7 8 9
3′
N M L K J I H G F E D C B

Figure S2

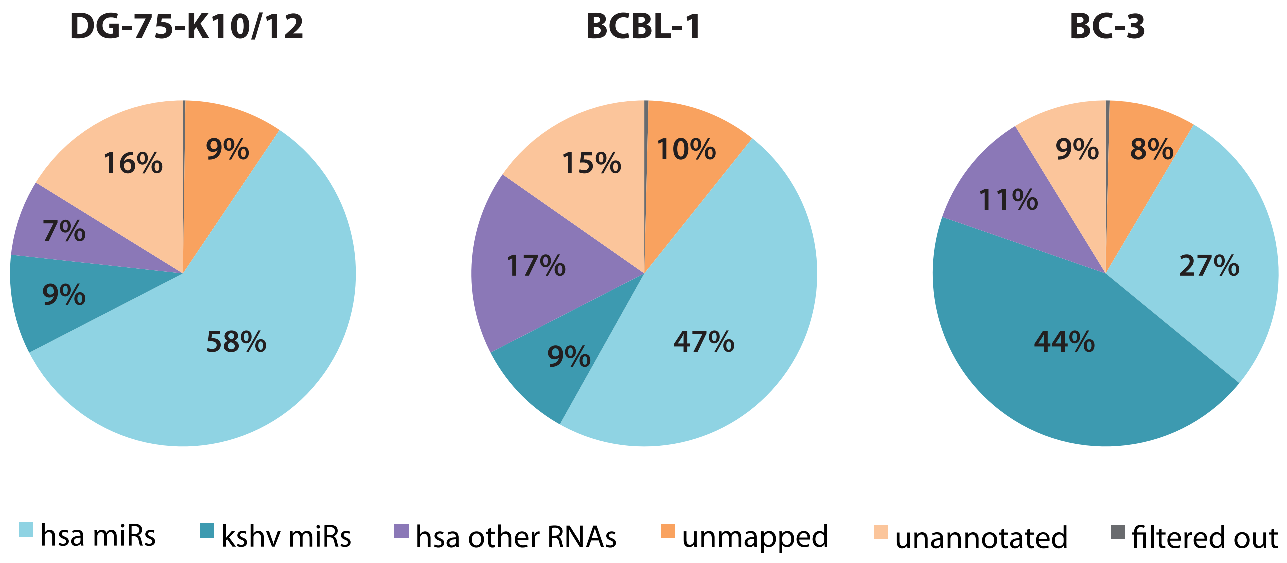
DG-75-K10/12
BCBL-1
BC-3
16%
9%
7%
9%
58%
15%
10%
17%
9%
47%
9%
8%
11%
27%
44%
hsa miRs
kshv miRs
hsa other RNAs
unmapped
unannotated
filtered out

Figure S3

```
                                     10        20        30        40        50

NC_009333.1   122282 TAAAACAGGAAGCGGGTTGGACTGGCAGGGTGGAGGAAGGATGTGGGGGTGGGGGGACG 122223
DG-75-K10/12         TAAAACAGGAAG-GGGTTGGAATGGCAGGGTGGAGGAAGGATGTGGGGGCGGGGGGACG
BCBL-1               TAAAACAGGAAGCGGGTTGGACTGGCAGGGTGGAGGAAGGATGTGGGGGCGGGGGGACG
BC-3                 TAAAACAGGAAGCGGGTTGGACTGGCAGGGTGGAGGAAGGATGTGGGGGTGGGGGGACG
                                 *        *                            *

              122222 AGGAAAAAGTACGCGGTTGTTTACGCAGGGTGCGGTGCTGCCCAGGACGGCCGGATGCGG 122163
                     AGGAAAAAGTACGCGGTTGTTTACGCAGGGTGCGGTGCTGCCCAGGACGGCCGGATGCGG
                     AGGAAAAAGTACGCGGTTGTTTACGCAGGGTGCGGTGCTGCCCAGGACGGCCGGATGCGG
                     AGGAAAAAGTACGCGGTTGTTTACGCAGGGTGCGGTGCTGCCCAGGACGGCCGGATGCGG

                                miR-K1-5p                           miR-K1-3p
              122162 GCGATTACAGGAAACTGGGTGTAAGCTGTACATAATCCCCGGCAGCACCTGTTTCCTGCA 122103
                     GCGATTACAGGAAACTGGGTGTAAGCTGTACATAATCCCCGGCAGCACCTGTTTCCTGCA
                     GCGATTACAGGAAACTGGGTGTAAGCTGTACATAATCCCCGGCAGCACCTGTTTCCTGCA
                     GCGATTACAGGAAACTGGGTGTAAGCTGTACATAATCCCCGGCAGCACCTGTTTCCTGCA

              122102 ACCCTCGTGTTTGGGCAAAACACATCCGCTGCCACCTGCGTGTTCCCGGCGCGAAAGTCC 122043
                     ACCCTCGTGTTTGGGCAAAACACATCCGCTGCCACCTGCGTGTTCCCGGCGCGAAAGTCC
                     ACCCTCGTGTTTGGGCAAAACACATCCGCTGCCACCTGCGTGTTCCCGGCGCGAAAGTCC
                     ACCCTCGTGTTTGGGCAAAACACATCCGCTGCCACCTGCGTGTTCCCGGCGCGAAAGTCC

                                                                      miR-K2-5p
              122042 GTCAACCAGATTTAAGATTAAGATTATAATGGGTCTACTTCGCTAACTGTAGTCCGGGTC 121983
                     GTCAACCAGATTTAAGATTAAGATTATAATGGGTCTACTTCGCTAACTGTAGTCCGGGTC
                     GTCAACCAGATTTAAGATTAAGATTATAATGGGTCTACTTCGCTAACTGTAGTCCGGGTC
                     GTCAACCAGATTTAAGATTAAGATTATAATGGGTCTACTTCGCTAACTGTAGTCCGGGTC

                                                       miR-K2-3p
              121982 GATCTGAGCCATTGAAGCAAGCTTCCAGATCTTCCAGGGCTAGAGCTGCCGCGGTGACAC 121923
                     GATCTGAGCCATTGAAGCAAGCTTCCAGATCTTCCAGGGCTAGAGCTGCCGCGGTGACAC
                     GATCTGAGCCATTGAAGCAAGCTTCCAGATCTTCCAGGGCTAGAGCTGCCGCGGTGACAC
                     GATCTGAGCCATTGAAGCAAGCTTCCAGATCTTCCAGGGCTAGAGCTGCCGCGGTGACAC

              121922 CAATTATAGAAATTTGGTCCCCAAACTCCCAACCAACGCAACAGCTACAATGCCTGTAAT 121803
                     CAATTATAGAAATTTGGTCCCCAAACTCCCAACCAACGCAACAGCTACAATGCCTGTAAT
                     CAATTATAGAAATTTGGTCCCCAAACTCCCAACCAACGCAACAGCTACAATGCCTGTAAT
                     CAATTATAGAAATTTGGTCCCCAAACTCCCAACCAACGCAACAGCTACAATGCCTGTAAT

                                 miR-K3-5p                             miR-K3-3p
              121862 GGGCTATCACATTCTGAGGACGGCAGCGACGTGTGTCTAACGTCAACGTCGCGGTCACAG 121743
                     GGGCTATCACATTCTGAGGACGGCAGCGACGTGTGTCTAACGTCAACGTCGCGGTCACAG
                     GGGCTATCACATTCTGAGGACGGCAGCGACGTGTGTCTAACGTCAACGTCGCGGTCACAG
                     GGGCTATCACATTCTGAGGACGGCAGCGACGTGTGTCTAACGTCAACGTCGCGGTCACAG

              121802 AATGTGACACCCCTCCAGGTCCAAGCGACGAACCGCCCGTGGCCACAACGCCGCCTGGTG 121683
                     AATGTGACACCCCTCCAGGTCCAAGCGACGAACCGCCCGTGGCCACAACGCCGCCTGGTG
                     AATGTGACACCCCTCCAGGTCCAAGCGACGAACCGCCCGTGGCCACAACGCCGCCTGGTG
                     AATGTGACACCCCTCCAGGTCCAAGCGACGAACCGCCCGTGGCCACAACGCCGCCTGGTG

                                           miR-K4-5p
              121742 GAACCGGGCAGTATAACTAGCTAAACCGCAGTACTCTAGGGCATTCATTTGTTACATAGA 121623
                     GAACCGGGCAGTATAACTAGCTAAACCGCAGTACTCTAGGGCATTCATTTGTTACATAGA
                     GAACCGGGCAGTATAACTAGCTAAACCGCAGTACTCTAGGGCATTCATTTGTTACATAGA
                     GAACCGGGCAGTATAACTAGCTAAACCGCAGTACTCTAGGGCATTCATTTGTTACATAGA

                         miR-K4-3p
              121682 ATACTGAGGCCTAGCTGATTATACTACCTCCGTCCAGCAGTCGACACGGATGTCTACGCC 121563
                     ATACTGAGGCCTAGCTGATTATACTACCTCCGTCCAGCAGTCGACACGGATGTCTACGCC
                     ATACTGAGGCCTAGCTGATTATACTACCTCCGTCCAGCAGTCGACACGGATGTCTACGCC
                     ATACTGAGGCCTAGCTGATTATACTACCTCCGTCCAGCAGTCGACACGGATGTCTACGCC

                                                                       miR-K5-5p
              121622 TCTCAAACCTCGTGGGCACGGCGTCATGACTAAGGGGGAGTTTGACCTAGGTAGTCCCTA
                     TCTCAAACCTCGTGGGCACGGCGTCATGACTAAGGGGGAGTTTGACCTAGGTAGTCCCTA
                     TCTCAAACCTCGTGGGCACGGCGTCATGACTAAGGGGGAGTTTGACCTAGGTAGTCCCTA
                     TCTCAAACCTCGTGGGCACGGCGTCATGACTAAGGGGGAGTTTGACCTAGGTAGTCCCTA
```

```
                        10        20        30        40        50
                 miR-K5-5p                            miR-K5-3p
NC_009333.1    121562 GTGCCCTAAGGGTCTACATCAAGCACTTAGGATGCCTGGAACTTGCCGGTCAAGCCCGCT 121503
DG-75-K10/12          GTGCCCTAAGGGTCTACATCAAGCACTTAGGATGCCTGGAACTTGCCGGTCAAGCCCGCT
BCBL-1                GTGCCCTAAGGGTCTACATCAAGCACTTAGGATGCCTGGAACTTGCCGGTCAAGCCCGCT
BC-3                  GTGCCCTAAGGGTCTACATCAAGCACTTAGGATGCCTGGAACTTGCCGGTCAAGCCCGCT

               121502 CATAAGACCATAACCTGGTGCCACAAAAACCTATGCGGGTCCAGCAAATGCTACCTTGGC 121443
                      CATAAGACCATAACCTGGTGCCACAAAAACCTATGCGGGTCCAGCAAATGCTACCTTGGC
                      CATAAGACCATAACCTGGTGCCACAAAAACCTATGCGGGTCCAGCAAATGCTACCTTGGC
                      CATAAGACCATAACCTGGTGCCACAAAAACCTATGCGGGTCCAGCAAATGCTACCTTGGC

               121442 AACACGTAAAAGATTTGGTCCCTGAAAGGGTCCGAAAACCACAACGTTATTTAGAACCAC 121383
                      AACACGTAAAAGATTTGGTCCCTGAAAGGGTCCGAAAACCACAACGTTATTTAGAACCAC
                      AACACGTAAAAGATTTGGTCCCTGAAAGGGTCCGAAAACCACAACGTTATTTAGAACCAC
                      AACACGTAAAAGATTTGGTCCCTGAAAGGGTCCGAAAACCACAACGTTATTTAGAACCAC

               121382 GGCAATCTCACCAAAAGGGGGTCCGTAGATGTAAATACAGCAAATAACAGGGTAAAAAAA 121323
                      GGCAATCTCACCAAAAGGGGGTCCGTAGATGTAAATACAGCAAATAACAGGGTAAAAAAA
                      GGCAATCTCACCAAAAGGGGGTCCGTAGATGTAAATACAGCAAATAACAGGGTAAAAAAA
                      GGCAATCTCACCAAAAGGGGGTCCGTAGATGTAAATACAGCAAATAACAGGGTAAAAAAA

               121322 TGAGTGGGCGGGGGCGGCAGTTTTACTTACTAAATCAGGGTAAACCCATAGTGACGGGGG 121263
                      TGAGTGGGCGGGGGCGGCAGTTTTACTTACAAAATCAGGGTAAACCCATAGTGACGGGGG
                      TGAGTGGGCGGGGGCGGCAGTTTTACTTACTAAATCAGGGTAAACCCATAGTGACGGGGG
                      TGAGTGGGCGGGGGCGGCAGTTTTACTTACTAAATCAGGGTAAACCCATAGTGACGGGGG

               121262 GAGGGTGCGTCACTAATTTGACACCATGCGGGAAACGCCAAACGGGCGATGGAAACTTGT 121203
                      GAGGGTGCGTCACTAATTTGACACCATGCGGGAAACGCCAAACGGGCGATGGAAACTTGT
                      GAGGGTGCGTCACTAATTTGACACCATGCGGGAAACGCCAAACGGGCGATGGAAACTTGT
                      GAGGGTGCGTCACTAATTTGACACCATGCGGGAAACGCCAAACGGGCGATGGAAACTTGT

               121202 CAACACTCCAAAAAAAATAGAAGGTTGGGTAACTGCGGGCTGGTGTCAACTTCTGTTAATT 121143
                      CAACACTCCAAAAAAAATAGAAGGTTGGGTAACTGCGGGCTGGTGTCAACTTCTGTTAATT
                      CAACACTCCAAAAAAAATAGAAGGTTGGGTAACTGCGGGCTGGTGTCAACTTCTGTTAATT
                      CAACACTCCAAAAAAAATAGAAGGTTGGGTAACTGCGGGCTGGTGTCAACTTCTGTTAATT

               121242 ATGAATAGTGTTTTTAAAGCGGGCGTTCGTAAGCGTCTGGATCGACACAAACCCTGCGCG 121083
                      ATGAATAGTGTTTTTAAAGCGGGCGTTCGTAAGCGTCTGGATCGACACAAACCCTGCGCG
                      ATGAATAGTGTTTTTAAAGCGGGCGTTCGTAAGCGTCTGGATCGACACAAACCCTGCGCG
                      ATGAATAGTGTTTTTAAAGCGGGCGTTCGTAAGCGTCTGGATCGACACAAACCCTGCGCG
                                           miR-K6-5p                miR-K6-3p
               121082 GGTTTAGAAAGACTTGTCCAGCAGCACCTAATCCATCGGCGGTGCGGGCTGATGGTTTTC 121023
                      GGTTTAGAAAGACTTGTCCAGCAGCACCTAATCCATCGGCGGTGCGGGCTGATGGTTTTC
                      GGTTTAGAAAGACTTGTCCAGCAGCACCTAATCCATCGGCGGTGCGGGCTGATGGTTTTC
                      GGTTTAGAAAGACTTGTCCAGCAGCACCTAATCCATCGGCGGTGCGGGCTGATGGTTTTC

               121022 GGGCTGTTGAGCGAGTCTTTTTATCTAGTCGCCCGTTATTGTTCTGTGTGTTCTTCATCA 120963
                      GGGCTGTTGAGCGAGTCTTTTTATCTAGTCGCCCGTTATTGTTCTGTGTGTTCTTCATCA
                      GGGCTGTTGAGCGAGTCTTTTTATCTAGTCGCCCGTTATTGTTCTGTGTGTTCTTCATCA
                      GGGCTGTTGAGCGAGTCTTTTTATCTAGTCGCCCGTTATTGTTCTGTGTGTTCTTCATCA

               120962 TTTCACCCACCGTCTCTCAATAGCCTTGGGAATCCTACCTCCACGCTCGCGTATGCCTCA 120903
                      TTTCACCCACCGTCTCTCAATAGCCTTGGGAATCCTACCTCCACGCTCGCGTATGCCTCA
                      TTTCACCCACCGTCTCTCAATAGCCTTGGGAATCCTACCTCCACGCTCGCGTATGCCTCA
                      TTTTACCCACCGTCTCTCAATAGCCTTGGGAATCCTACCTCCACGCTCGCGTATGCCTCA
                         *
                                      miR-K11-5p                       miR-K11-3p
               120902 TGCGAGGTTCGCTTTGGTCACAGCTTAAACATTTCTAGGGCGGTGTTATGATCCTTAATG 120843
                      TGCGAGGTTCGCTTTGGTCACAGCTTAAACATTTCTAGGGCGGTGTTATGATCCTTAATG
                      TGCGAGGTTCGCTTTGGTCACAGCTTAAACATTTCTAGGGCGGTGTTATGATCCTTAATG
                      TGCGAGGTTCGCTTTGGTCACAGCTTAAACATTTCTAGGGCGGTGTTATGATCCTTAATG
```

```
                    10        20        30        40        50
                miR-K11-3p
NC_009333.1   120842 CTTAGCCTGTGTCCGATGCGGCCCCGTGCCTTGCGGAAGCGCGGGCGGGTTCCCGACCTT 120783
DG-75-K10/12         CTTAGCCTGTGTCCGATGCGGCCCCGTGCCTTGCGGAAGCGCGGGCGGGTTCCCGACCTT
BCBL-1               CTTAGCCTGTGTCCGATGCGGCCCCGTGCCTTGCGGAAGCGCGGGCGGGTTCCCGACCTT
BC-3                 CTTAGCCTGTGTCCGATGCGGCCCCGTGCCTTGCGGAAGCGCGGGCGGGTTCCCGACCTT

              120782 CACGGCGGCAATTTTTGTCGTATGCGCCTCCCCTCGCCTACAAGTCCAATACCCATGAGA 120723
                     CACGGCGGCAATTTTTGTCGTATGCGCCTCCCCTCGCCTACAAGTCCAATACCCATGAGA
                     CACGGCGGCAATTTTTGTCGTATGCGCCTCCCCTCGCCTACAAGTCCAATACCCATGAGA
                     CACGGCGGCAATTTTTGTCGTATGCGCCTCCCCTCGCCTACAAGTCCAATACCCATGAGA

              120722 CCACCGTACGCGTGCCCACCGATGAGATACCACGCAGCCGCGCATATTGGCGTTGAGCGC 120663
                     CCACCGTACGCGTGCCCACCGATGAGATACCACGCAGCCGCGCATATTGGCGTTGAGCGC
                     CCACCGTACGCGTGCCCACCGATGAGATACCACGCAGCCGCGCATATTGGCGTTGAGCGC
                     CCACCGTACGCGTGCCCACCGATGAGATACCACGCAGCCGCGCATATTGGCGTTGAGCGC
                  miR-K7-5p                              miR-K7-3p
              120662 CACCGGACGGGGATTTATGCTGTATCTTACTACCATGATCCCATGTTGCTGGCGCTCACG 120603
                     CACCGGACGGGGATTTATGCTGTATCTTACTACCATGATCCCATGTTGCTGGCGCTCACG
                     CACCGGACGGGGATTTATGCTGTATCTTACTACCATGATCCCATGTTGCTGGCGCTCACG
                     CACCGGACGGGGATTTATGCTGTATCTTACTACCATGATCCCATGTTGCTGGCGCTCACG

              120602 GCCCGTGTGCCAGCCGCCTGGACGTCGAAAGCACGCAAAGCTGAACGTTCGGGCTTAGTG 120543
                     GCCCGTGTGCCAGCCGCCTGGACGTCGAAAGCACGCAAAGCTGAACGTTCGGGCTTAGTG
                     GCCCGTGTGCCAGCCGCCTGGACGTCGAAAGCACGCAAAGCTGAACGTTCGGGCTTAGTG
                     GCCCGTGTGCCAGCCGCCTGGACGTCGAAAGCACGCAAAGCTGAACGTTCGGGCTTAGTG

              120542 TAGCGCGAACCATACGACCTTGGTCCCCTGGCCAAAGAGGGGCGCAAAACAATTGTGGGT 120483
                     TAGCGCGAACCATACGACCTTGGTCCCCTGGCCAAAGAGGGGCGCAAAACAATTGTGGGT
                     TAGCGCGAACCATACGACCTTGGTCCCCTGGCCAAAGAGGGGCGCAAAACAATTGTGGGT
                     TAGCGCGAACCATACGACCTTGGTCCCCTGGCCAAAGAGGGGCGCAAAACAATTGTGGGT

              120482 GGCTATCCTGCCCAGAGTCCCATATATTCTGGTGGGTAGCTAACCTGCCCAGGACGTCCA 120423
                     GGCTATCCTGCCCAGAGTCCCATATATTCTGGTGGGTAGCTAACCTGCCCAGGACGTCCA
                     GGCTATCCTGCCCAGAGTCCCATATATTCTGGTGGGTAGCTAACCTGCCCAGGACGTCCA
                     GGCTATCCTGCCCAGAGTCCCATATATTCTGGTGGGTAGCTAACCTGCCCAGGACGTCCA

              120422 AATATGCCAAATGGTACCTTAGTGTCTACTTTGCATATTTGGAACCCGTGTATCCATGTA 120363
                     AATATGCCAAATGGTACCTTAGTGTCTACTTTGCATATTTGGAACCCGTGTATCCATGTA
                     AATATGCCAAATGGTACCTTAGTGTCTACTTTGCATATTTGGAACCCGTGTATCCATGTA
                     AATATGCCAAATGGTACCTTAGTGTCTACTTTGCATATTTGGAACCCGTGTATCCATGTA

              120362 TGGTTATCGTGCCCAGGGTGCCCAAATTGCTCAATAATGAAAATTCCATTCTAGCGCTGG 120303
                     TGGTTATCGTGCCCAGGGTGCCCAAATTGCTCAATAATGAAAATTCCATTCTAGCGCTGG
                     TGGTTATCGTGCCCAGGGTGCCCAAATTGCTCAATAATGAAAATTCCATTCTAGCGCTGG
                     TGGTTATCGTGCCCAGGGTGCCCAAATTGCTCAATAATGAAAATTCCATTCTAGCGCTGG
                                                                     miR-K8-5p
              120302 GGTGACAAAGCATGCACTGGAAATCGGCCAAGTCAGCGCAGCGCGCGCACTCCCTCACTA 120243
                     GGTGACAAAGCATGCACTGGAAATCGGCCAAGTCAGCGCAGCGCGCGCACTCCCTCACTA
                     GGTGACAAAGCATGCACTGGAAATCGGCCAAGTCAGCGCAGCGCGCGCACTCCCTCACTA
                     GGTGACAAAGCATGCACTGGAAATCGGCCAAGTCAGCGCAGCGCGCGCACTCCCTCACTA
                                                    miR-K8-3p
              120242 ACGCCCCGCTTTTGTCTGTTGGAAGCAGCTAGGCGCGACTGAGAGAGCACGCGCGCGGCG 120183
                     ACGCCCCGCTTTTGTCTGTTGGAAGCAGCTAGGCGCGACTGAGAGAGCACGCGCGCGGCG
                     ACGCCCCGCTTTTGTCTGTTGGAAGCAGCTAGGCGCGACTGAGAGAGCACGCGCGCGGCG
                     ACGCCCCGCTTTTGTCTGTTGGAAGCAGCTAGGCGCGACTGAGAGAGCACGCGCGCGGCG

              120182 CCCACGCCGGCGCCTGGCACGCGGCCAACATAAAGTGTGGATGGCCCTGCTATCTGACTA 120123
                     CGCACGCCGGCGCCTGGCACGCGGCCAACATAAAGTGTGGATGGCCCTGCTATCTGACTA
                     CCCACGCCGGCGCCTGGCACGCGGCCAACATAAAGTGTGGATGGCCCTGCTATCTGACTA
                     CCCACGCCGGCGCCTGGCACGCGGCCAACATAAAGTGTGGATGGCCCTGCTATCTGACTA
                      *
```

```
                             10        20        30        40        50

NC_009333.1   120122 ATTTGCATGCGCTAATCCTTTTATGTGCATAAATTATGTAGATGAGGAGTCGCGCATGCG 120063
DG-75-K10/12         ATTTGCATGCGCTAATCCTTTTATGTGCATAAATTATGTAGATGAGGAGTCGCGCATGCG
BCBL-1               ATTTGCATGCGCTAATCCTTTTATGTGCATAAATTATGTAGATGAGGAGTCGCGCATGCG
BC-3                 ATTTGCATGCGCTAATCCTTTTATGTGCATAAATTATGTAGATGAGGAGTCGCGCATGCG

              120062 CAGAAAAATTCAGAGCGCCGGGTGCACGGGGTCACCTCCAGGTCACGCCGCTAGGTGGGA 120003
                     CAGAAAAATTCAGAGCGCCGGGTGCACGGGGTCACCTCCAGGTCACGCCGCTAGGTGGGA
                     CAGAAAAATTCAGAGCGCCGGGTGCACGGGGTCACCTCCAGGTCACGCCGCTAGGTGGGA
                     CAGAAAAATTCAGAGCGCCCGGGTGCACGGGGTCACCTCCAGGTCACGCCGCTAGGTGGG
                                        *

              120002 CCGTGAGCGACTCGAAAAATTATAATTTTTGGCCATTTTATGGGCGCCGCCATCTTGAAT 119943
                     CCGTGAGCGACTCGAAAAATTATAATTTTTGGCCATTTTATGGGCGCCGCCATCTTGAAT
                     CCGTGAGCGACTCGAAAAATTATAATTTTTGGCCATTTTATGGGCGCCGCCATCTTGAAT
                     ACCGTGAGCGACTCGAAAAATTATAATTTTTGGCCATTTCATGGGCGCCGCCATCTTGAA
                                                            *

              119942 TTGCTAATCCCCCGTAATCCTCTGCCCCTCTCCCATT-GGTCCGCCGGCCCGTCAATCAA 119884
                     TTGCTAATCCCCCGTAATCCTCTGCCCCTCTCCCATT-GGTCCGCCGGCCCGTCAATCAA
                     TTGCTAATCCCCCGTAATCCTCTGCCCCTCTCCCATT-GGTCCGCCGGCCCGTCAATCAA
                     TTTGCTAATCCCCCGTAATCCTCTGCCCCTCTCCCATTGGTCCGCCGGCCCGTCAATCAA
                                                          *

              119883 AGTTTTCCGAGCCGCCATTGGCCCATTCGGCCGACCAATCTCGTTCGAGCTAGGCGACCG 119824
                     AGTTTTCCGAGCCGCCATTGGCCCATTCGGCCGACCAATCCCGTTCGAGCTAGGCGACCG
                     AGTTTTCCGAGCCGCCATTGGCCCATTCGGCCGACCAATCCCGTTCGAGCTAGGCGACCG
                     AGTTTTCCGAGCAGCCATTGGCCCATTCGGCCGACCAATCCCGTTCGAGCTAGGCGACCG
                                 *                           *

              119823 CGCCATTCCATTGGACGCCCCAGCCGTCAATCAAATTCGGAGGCCTCCCATTGGCCCCTA 119764
                     CGCCATTCCATTGGACGCCCCAGCCGTCAATCAAATTCGGAGGCCTTCCATTGGCCCCTA
                     CGCCATTCCATTGGACGCCCCAGCCGTCAATCAAATTCGGAGGCCTCCCATTGGCCCCTA
                     CGCCATTCCATTGGACGCCCCAGCCGTCAATCAAATTCGGAGGCCTCCCATTGGCCCCTA
                                                                   *

              119763 TCCCTAGAACTCCCAAGCTGATTGGCCCACAGCGGGAACCAATCAGCGATTAGAGTTTTG 119704
                     TCCCTAGAACTCCCAAGCTGATTGGCCCACAGCGGGAACCAATCAGCGATTAGAGTTTTG
                     TCCCTAGAACTCCCAAGCTGATTGGCCCACAGCGGGAACCAATCAGCGATTAGAGTTTTG
                     TCCCTAGAACTCCCAAGCTGATTGGCCCACAGCGGGAACCAATCAGCGATTAGAGTTTTG

              119703 TTTTGATTTTTCCTATATATATATATATAATCCTTTAATCCTAGCGCAGCTGAGTCATCG 119644
                     TTTTGATTTTTCCTATATATATATATAAAATCCTTTAATCCTAGCGCAGCTGAGTCATCG
                     TTTTGATTTTTCCTATATATATATATAAAATCCTTTAATCCTAGCGCAGCTGAGTCATCG
                     TTTTGATTTTTCCTATATATATATATATAATCCTTTAATCCTAGCGCAGCTGAGTCATCG
                                                *
                                                                miR-K9-5p
              119643 CAGCCCCTATTCCAGTAGGTATACCCAGCTGGGTCTACCCAGCTGCGTAAACCCCGCTGC 119584
                     CAGCCCCTATTCCAGTAGGTATACCCAGCTGGGTCTACCCAGCTGCGTAAACCCCGCTGC
                     CAGCCCCTATTCCAGTAGGTATACCCAGCTGGGTCTACCCAGCTGCGTAAACCCCGCTGC
                     CAGCCCCTATTCCAGTAGGTATACCCAGCTGGGTA-ATCCAG-TAGGTATACCCAGGTGG
                                                       ** *    * **   *    * *  *
                                      miR-K9-3p
              119583 GTAAACACAGCTGGGTATACGCAGCTGCGTAAACCCGGCTGGGTAAATCCAGCTG--TAA 119526
                     GTAAACACAGCTGGGTATACGCAGCTGCGTAAACCCGGCTGGGTAAATCCAGCTG--TAA
                     GTAAACACAGCTGGGTATACGCAGCTGCGTAAACCCGGCTGGGTAAATCCAGCTG--TAA
                     GTGAACCCAGCTGGGTATACCCAGCTGCGTAAACCCGGCTGGGTATACCCAGCTGCGTAA
                       *   *             *                        * *       **

              119525 TTCTACAATTAAACAAGGTAT 119505
                     TTCTACAATTAAACAAGGTAT
                     TTCTACAATTAAACAAGGTAT
                     TTCTACAATTAAACAAGGTAT
```

Figure S4

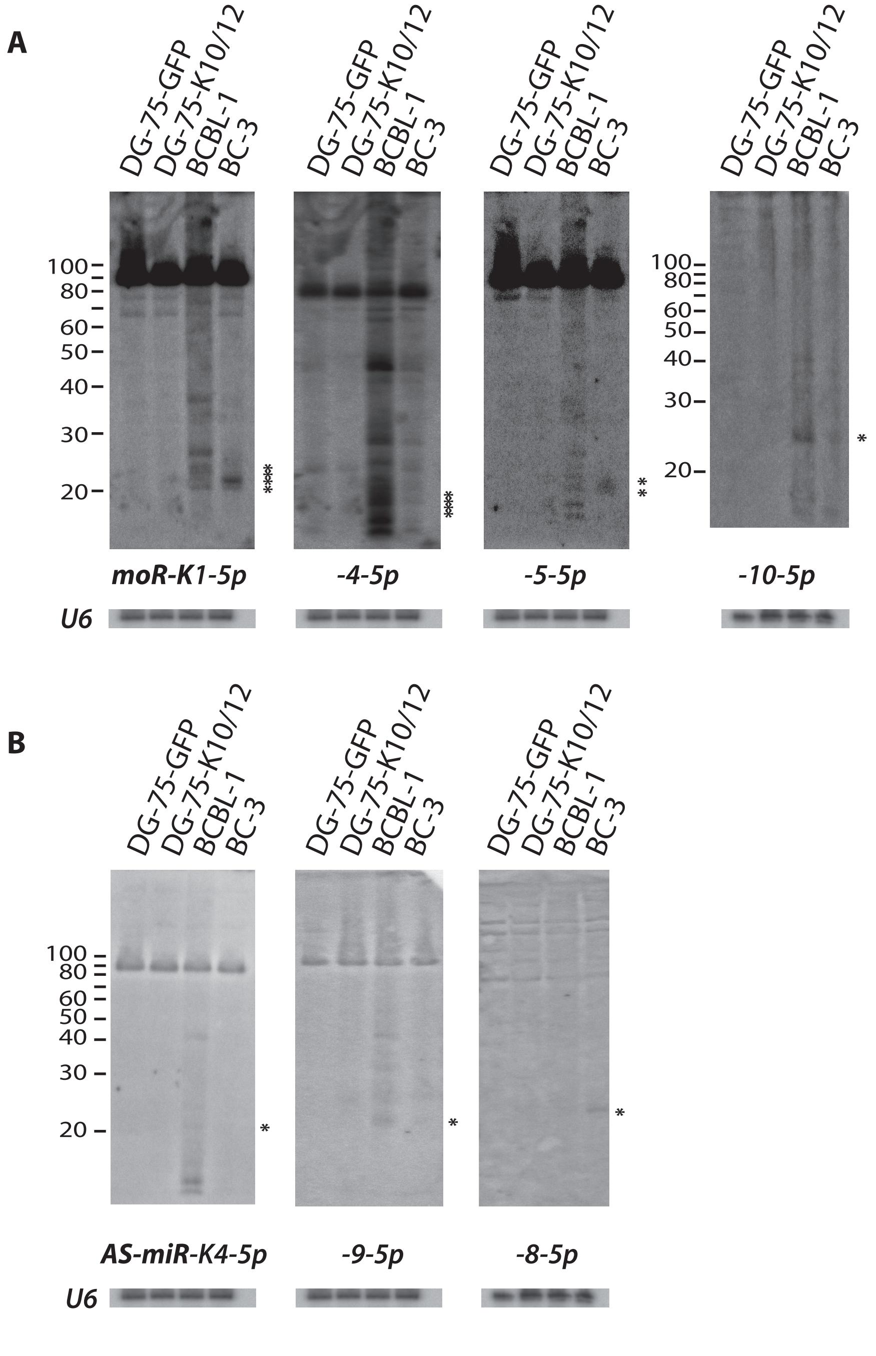
A
DG-75-GFP
DG-75-K10/12
BCBL-1
BC-3
100
80
60
50
40
30
20
moR-K1-5p
-4-5p
-5-5p
-10-5p
U6
B
AS-miR-K4-5p
-9-5p
-8-5p

Figure S5

Optimal stem-loop structures

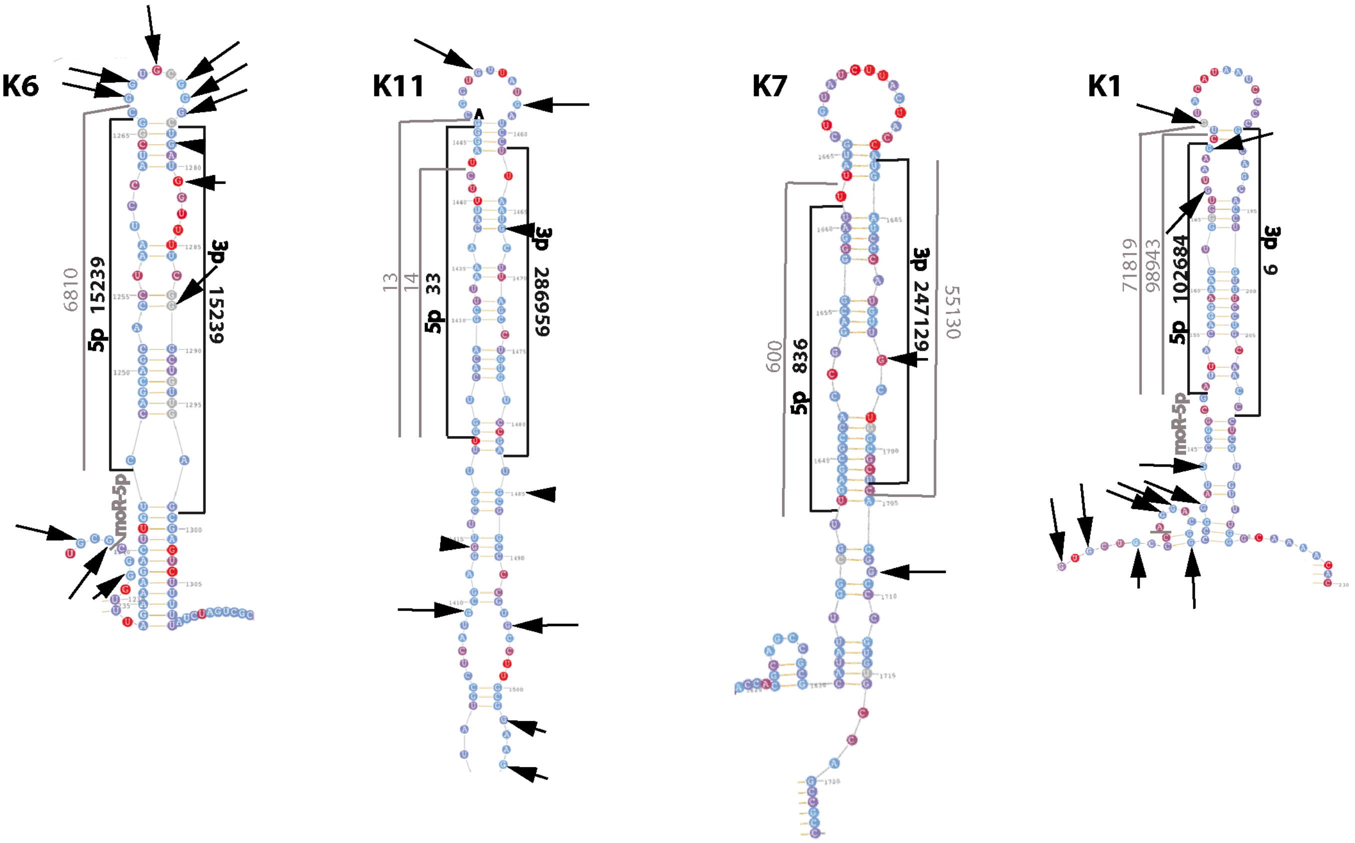

Sub-optimal stem-loop structures

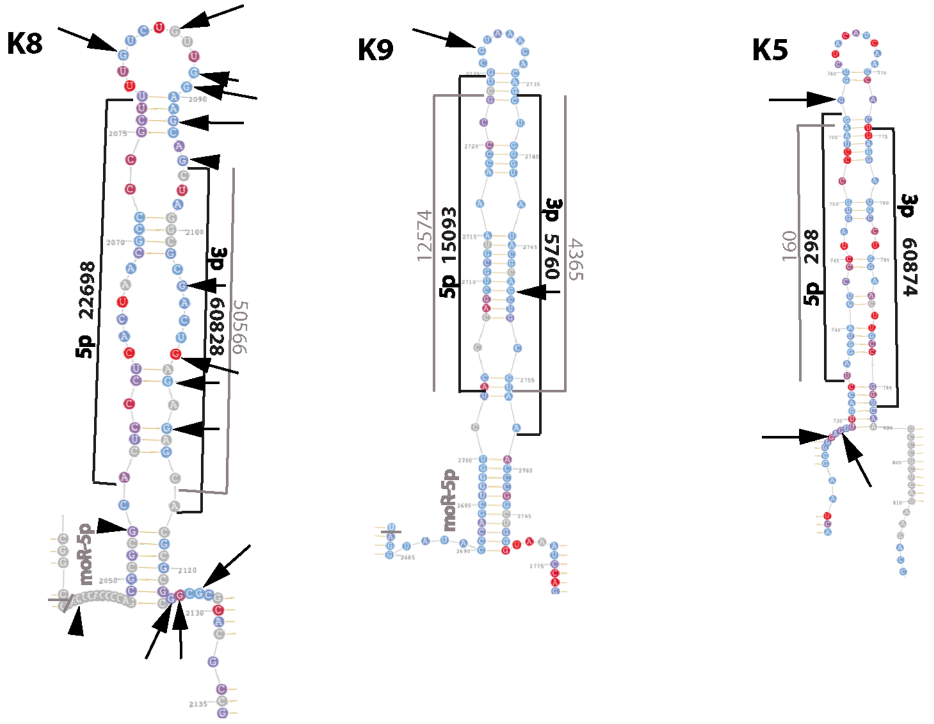

Figure S6

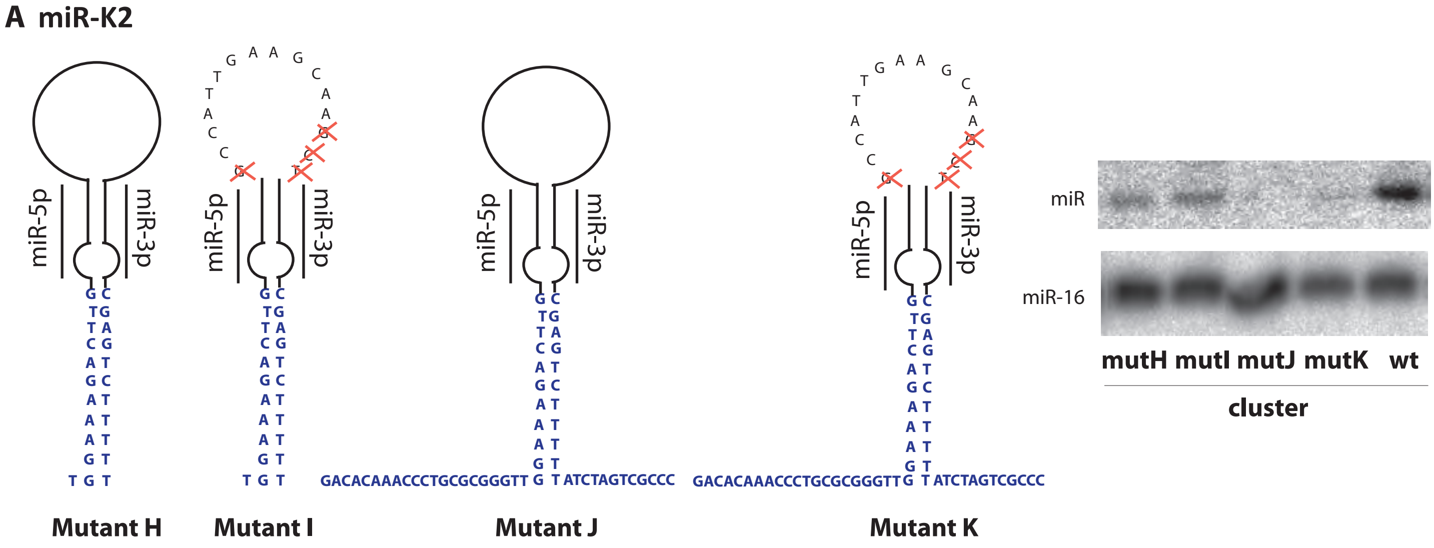
A miR-K2
miR-5p
miR-3p
GACACAAACCCTGCGCGGGTT G T ATCTAGTCGCCC
Mutant H
Mutant I
Mutant J
Mutant K
miR
miR-16
mutH mutI mutJ mutK wt
cluster

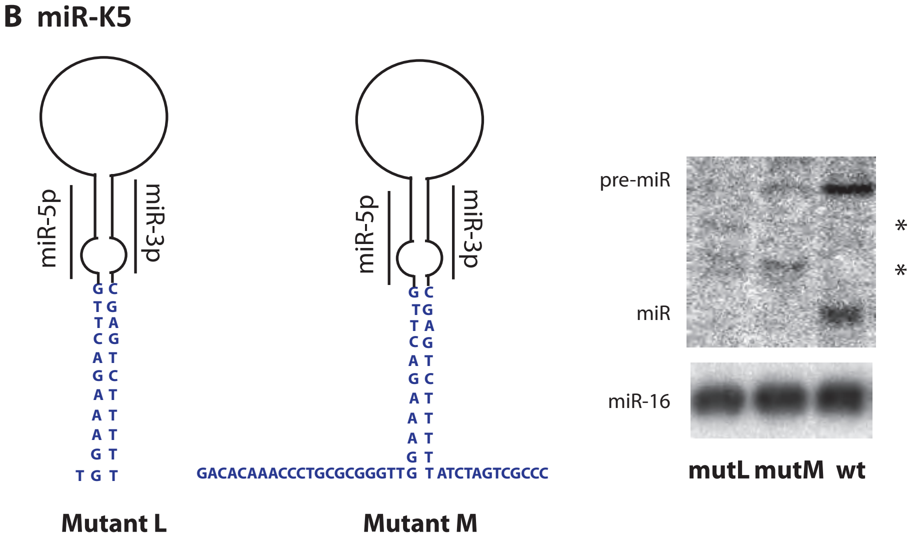
B miR-K5
miR-5p
miR-3p
GACACAAACCCTGCGCGGGTTG T ATCTAGTCGCCC
Mutant L
Mutant M
pre-miR
miR
miR-16
*
*
mutL mutM wt
